# Systolic blood pressure status modifies the associations between the triglyceride-glucose index and incident cardiovascular disease: a national cohort study in China

**DOI:** 10.1186/s12933-024-02227-w

**Published:** 2024-04-24

**Authors:** Weida Qiu, Anping Cai, Liwen Li, Yingqing Feng

**Affiliations:** Department of Cardiology, Hypertension Research Laboratory, Guangdong Cardiovascular Institute, Guangdong Provincial People’s Hospital, Guangdong Academy of Medical Sciences, Southern Medical University, No. 106, Zhongshan 2nd Road, Yuexiu District, Guangzhou, 510080 China

**Keywords:** Triglyceride-glucose index, Blood pressure, Hypertension, Cardiovascular disease

## Abstract

**Background:**

The triglyceride-glucose (TyG) index and blood pressure (BP) are correlated and serve as risk factors for cardiovascular disease (CVD). The potential impact of BP status on the association between the TyG index and CVD risk remains uncertain. This study aims to investigate the relationships between the TyG index and incident CVD in Chinese middle-aged and elderly adults, considering variations in BP status among participants.

**Methods:**

6558 participants (mean age: 58.3 (± 8.7) years; 46.0% were men) without prevalent CVD were recruited from the China Health and Retirement Longitudinal Study. Participants were divided into three groups according to their systolic blood pressure (SBP) levels (< 120mmHg, 120 ∼ 129mmHg, ≥ 130mmHg). The TyG index was computed as ln[triglyceride (mg/dl) * fasting blood glucose (mg/dl)/2]. The primary outcome was CVD (heart disease and stroke), and the secondary outcomes were individual CVD components. Cox regression models and restricted cubic splines were performed to investigate the associations between continuous and categorical TyG with CVD.

**Results:**

1599 cases of CVD were captured during 58,333 person-years of follow-up. Per 1-SD higher TyG index was associated with a 19% (HR: 1.19; 95% CI: 1.12, 1.27) higher risk for incident CVD, and the participants with the highest quartile of TyG index had a 54% (HR: 1.54; 95% CI: 1.29, 1.84) higher risk of CVD compared to those in the lowest quartile. SBP significantly modifies the association between the TyG index and CVD, with higher HRs for CVD observed in those with optimal and normal SBP. SBP partially mediated the associations between the TyG index with CVD. The results were generally consistent among participants with varying pulse pressure statuses rather than diastolic BP statuses.

**Conclusions:**

The associations between the TyG index and CVD were modified by BP status, with greater HRs for CVD observed among those who had SBP < 130mmHg. SBP can partially mediate the association between the TyG index with CVD, highlighting the importance of early screening for the TyG index to identify at risk of hypertension and CVD.

**Supplementary Information:**

The online version contains supplementary material available at 10.1186/s12933-024-02227-w.

## Introduction

According to a recent epidemiological report, cardiovascular disease (CVD) has ranked as the primary contributor to death worldwide. Albeit a 34.9% reduction in CVD mortality, the number of cardiovascular deaths has surged by 19.8 million in 2022 [1], which causes a major burden on human life quality, healthcare expenditure, and the social economy [2]. Among the overall risk factors for CVD, high systolic blood pressure (SBP) is the most critical agent for cardiovascular health [1, 2]. By 2022, hypertension has led to 256.9 per 100 thousand age-standardized CVD disability-adjusted life years worldwide [1]. Furthermore, according to the World Health Organization’s report, the estimated prevalence of hypertension has increased by 33%, and the prevalence is projected to rise alarmingly with the aging society [3].

Triglyceride-glucose (TyG) index is a reliable biochemical surrogate of insulin resistance among diabetic and non-diabetic individuals [4, 5]. Unlike other methods used to assess insulin resistance, the TyG index is a more convenient and reproducible alternative that does not necessitate fasting insulin quantification [6]. Mountains of evidence show that the TyG index independently and significantly predicts several CVDs and poor prognoses, including arterial stiffness [7], coronary artery disease [8], heart failure [9], stroke [10], and mortality [11, 12]. A more recent study found an additive interaction of TyG index and body mass index (BMI) on stroke among 8 231 Chinese middle-aged and elderly participants, with TyG index found to mediate over half of the total association [13], indicating that common cardiovascular factors may intertwine and modify their impact on cardiovascular risks.

By 2022, hypertension has led to 256.9 per 100 thousand age-standardized CVD disability-adjusted life years worldwide [1]. Furthermore, according to the World Health Organization’s report, the estimated prevalence of hypertension has increased by 33%, and the prevalence is projected to rise alarmingly with the aging society [3]. In addition, insulin resistance and BP are inextricably linked [14, 15]. What’s more, a recent Kailuan Study [16] along with several studies [15, 17] demonstrate that dual assessment of the cumulative TyG index and SBP provides significant insights into CVD prevention. Nevertheless, research on such issues is scarce to date, and it remains unclear whether the BP status modifies the relationship between the TyG index with CVD. Therefore, we believe that comprehensively depicting the effects of the TyG index on incident CVD among subjects with varying BP statuses is essential for precise prevention and shared-decision making.

Leveraging a national cohort from the China Health and Retirement Longitudinal Study (CHARLS), we aimed to 1) assess the associations between the TyG index with incident CVD among participants with different BP statuses, and (2) examine the potential mediating effect of BP on the associations between TyG with CVD among Chinese middle-aged and elderly adults.

## Methods

### Study participants

The CHARLS is an ongoing prospective cohort study that was launched in 2011, involving 17,708 participants from 28 provinces across mainland China [18]. A multistage, stratified, probability-proportional-to-size sampling method was employed to promise a nationally representative sample. Participants with ages ≥ 45 years and their spouses were eligible for the recruitment. Participants were enrolled after the written informed consent was obtained, with subsequent follow-up every two to three years until 2020. The CHARLS protocol was approved by the ethics review committee at Peking University (IRB00001052-11015).

The current study excluded participants with age < 45 years (*N* = 165), without data on TyG index (*N* = 6072) and BP (*N* = 1827), without a follow-up record in 2020 (*N* = 1639), with a history of CVD at baseline (stroke: *N* = 157; heart disease: *N* = 841), and with missing covariates at baseline (*N* = 449). A total of 6558 participants aged ≥ 45 years without prevalent CVD and missing any covariates were finally included in the analysis. (Supplemental Fig. 1)

### Assessment of BP and TyG index

Each recruited participant received a physical examination to collect BPs. Specifically, BPs were measured three times at 45-second intervals using the OmronTM HEM-7200 Monitor (Omron (Dalian) Co., LTD., Dalian, China) by trained staff. The average values of three measurements were recorded. Anthropometric measurements, including height, weight, and waist circumference, were also performed during the examination following the cohort profile [18]. Venous blood samples were drawn from the participants after fasting for at least 8 h and stored at -80℃. Triglyceride (TG) and serum glucose levels were analyzed at the central laboratory using the enzyme colorimetric assay. The TyG index was computed as ln[TG (mg/dl) * fasting blood glucose (FBG) (mg/dl)/2]. Other laboratory tests, including hemoglobin, hemoglobin A1c (HbA1c), uric acid, total cholesterol (TC), low density lipoprotein cholesterol (LDL-C), high density lipoprotein cholesterol (HDL-C), creatinine, and C-reactive protein, were simultaneously examined at the central laboratory.

### Outcomes ascertainment

The primary outcome of the present study was incident CVD (a composite of heart disease and stroke (both ischemic and hemorrhagic)), and the secondary outcomes were the individual CVD components. The endpoints were captured during a personal interview. The ascertainment of CVD was defined as a self-reported diagnosis or self-reported treatment for CVD. According to the cohort profile, the heart disease included the following conditions: coronary heart disease or angina, heart attack, heart failure, and other heart problems. The follow-up data used to collect the events were obtained from the follow-up surveys conducted in 2013, 2015, 2018, and 2020. Follow-up duration was calculated by subtracting the date of enrollment from the date of CVD events or death, or the last follow-up date (December 2020).

### Covariates

Other baseline covariates were collected from the first survey conducted in 2011. Data on demographics (i.e., age and sex), socioeconomic status (i.e., marriage, educational attainment, and residential area), and comorbid conditions (i.e., smoking status, alcohol drinking status, hypertension, diabetes mellitus (DM), dyslipidemia, chronic lung disease, and chronic kidney disease) were collected using a structured questionnaire through a face-to-face interview. The baseline comorbidities were self-reported. Specifically, each individual was asked the following questions: “Have you been diagnosed with each corresponding comorbidity?” If so, he/she would be asked for having been receiving any treatment for the corresponding condition. The physical examination and laboratory tests were performed as stated above, and the modified diet of renal disease formula was employed to calculate the estimated glomerular filtration rate (eGFR) [19].

### Statistical analysis

Participants were divided into three groups based on their SBP levels according to the guideline recommendations [20, 21], namely, individuals with optimal SBP (< 120mmHg), individuals with normal SBP (between 120 and 129mmHg), and individuals with high SBP (≥ 130mmHg). Baseline characteristics were presented as means and standard deviations (SDs) and frequency with percentage, accordingly. The normality of the continuous variables was assessed using the Kolmogorov-Smirnov test, and the skewed distribution variable (i.e., C-reactive protein (CRP)) was presented as medians and interquartile ranges (IQRs). The difference between BP groups was compared using the One-way ANOVA test, the Kruskal–Wallis H-test (for skewed distribution variable), and the Chi-square test.

The multivariate Cox regression model was conducted to estimate the associations between the TyG index (continuous and categorized as quartiles) and incident CVD among overall participants as well as in three SBP groups, with full adjustment for demographics (i.e., age, sex, marital status (single or married), educational attainment (less than high school or not), residence (urban or rural)), physical examination (i.e., SBP, pulse, BMI, waist circumference), laboratory (i.e., hemoglobin, LDL-C, HDL-C, uric acid, eGFR, CRP, HbA1c), self-reported comorbidities (i.e., smoking status (current or non-current), drinking status (current or non-current), hypertension (yes or no), anti-hypertensive treatment (yes or no), DM (yes or no), anti-diabetic treatment (yes or no), dyslipidemia (yes or no), lipid-lowering treatment (yes or no), and chronic lung disease (yes or no)). Hazard ratios (HRs) and 95% confidence intervals (CIs) were reported. Given that multiple covariates were adjusted in the final model, the variance inflation factor (VIF) was used to check the multicollinearity between covariates, and VIF > 5 was considered indicative of collinearity and excluded from the model. The TyG index-by-SBP group interactions were tested and included in the multi-adjusted models using the likelihood ratio tests, and the relative HRs with SBP group increment were also presented. The flexible relationships between the TyG index and the risk probability of incident CVD among varying SBP groups were visualized using restricted cubic spline (RCS) analysis based on the fully adjusted Poisson regression models. RCS based on the Cox regression model was also conducted to determine the potential linear or nonlinear association between the TyG index and CVD among overall participants and individuals with varying SBP statuses, with adjustments for the aforementioned covariates.

### Additional analysis

After identifying that the CVD risk increased more steeply with a higher TyG index among participants who had SBP less than 130mmHg, and the risk even exceeded those with the highest SBP (≥ 130mmHg) level, rather than the participants with both high SBP and high TyG index holding the highest risk for CVD, we speculate that SBP might be a significant mediator of the relationships between TyG index with incident CVD, which might be an underlying explanation for our observations. Therefore, the Preacher’s Sobel test with a bootstrap method with 500-time resamples [22] was performed to estimate the mediation effects of SBP on the associations between the TyG index with CVD, with adjustment for the identical covariates included in the Cox regression models. The proportion of mediation was computed as the indirect effect dividing the total effect, and the CIs were calculated based on bias-corrected bootstrapping. To reduce the bias of reverse causation, we used the SBP that was recorded in the 2015 survey rather than the 2011 survey. In addition to SBP, mediator analyses between DBP and PP with CVD were also performed.

Except for the SBP statuses, we also stratified participants according to their diastolic blood pressure (DBP) and pulse pressure (PP) levels. Based on the DBP level, subjects were divided into three groups (i.e., individuals with DBP < 80mmHg, individuals with DBP between 80 and 90mmHg, and individuals with DBP > 90mmHg). Based on the PP median (51mmHg), participants were divided into two groups. To comprehensively characterize the associations between the TyG index and CVD, we repeated the above analysis among participants with varying DBP and PP statuses. In addition, we also evaluate the differential associations between the TyG index and individual CVD components in participants with different BP statuses. Finally, a sensitivity analysis was performed after excluding participants with self-reported DM, with HbA1c ≥ 6.5% or FBG ≥ 126 mg/dL.

All analyses were carried out using Stata 15.0 (StataCorp, College Station, TX, USA) and R version 4.1.2 (R Foundation for Statistical Computing, Vienna, Austria), and statistical significance was defined as two-sided *p*-values < 0.05.

## Results

### Baseline characteristics of study participants

A total of 6558 participants were included in the analysis, with a mean age of 58.3 (± 8.7) years and 46.0% were men. Among them, 37.1% (*N* = 2434) had SBP < 120mmHg, 20.2% (*N* = 1324) had SBP between 120 and 129mmHg, and 42.7% (*N* = 2800) had SBP > 130mmHg, with a mean SBP of 129.2mmHg and a mean TyG index of 8.7. Participants with a higher level of SBP tended to be older, more likely to be single, had lower educational attainment, and had higher prevalences of DM and dyslipidemia. In addition, they also had higher levels of BMI, waist circumference, and lipid profiles. (Table 1)

**Table 1:** Baseline characteristics comparison stratified by the systolic blood pressure status

Variables	Overall (*N* = 6558)	SBP			*P*-value
		< 120mmHg (*N* = 2434)	120 ∼ 129mmHg (*N* = 1324)	> 130mmHg (*N* = 2800)	
**Demographic**					
Age (years)	58.3 ± 8.7	56.2 ± 7.9	57.3 ± 8.1	60.6 ± 9.1	< 0.001
Male, n(%)	3017 (46.0)	1052 (43.2)	664 (50.2)	1301 (46.5)	< 0.001
Married, n(%)	5884 (86.1)	2251 (92.5)	1222 (92.3)	2411 (86.1)	< 0.001
Rural residence, n(%)	5464 (83.3)	2035 (83.6)	1074 (81.1)	2355 (84.1)	0.049
Educational attainment < High school, n(%)	5919 (92.2)	2167 (89.0)	1171 (88.4)	2581 (92.2)	< 0.001
**Physical examination**					
SBP (mmHg)	129.2 ± 20.8	109.6 ± 7.2	124.8 ± 2.9	148.4 ± 16.2	< 0.001
DBP (mmHg)	75.4 ± 11.9	66.0 ± 7.5	74.8 ± 7.1	83.8 ± 10.6	< 0.001
Pulse (beat per minute)	72.1 ± 10.1	71.9 ± 9.9	72.3 ± 9.9	72.1 ± 10.3	0.444
Body mass index (kg/m^2^)	23.5 ± 3.8	22.7 ± 3.5	23.5 ± 3.7	24.1 ± 4.1	< 0.001
Waist circumference (cm)	84.0 ± 12.3	81.0 ± 12.1	84.1 ± 11.8	86.4 ± 12.2	< 0.001
**Laboratory**					
Hemoglobin (g/dL)	14.4 ± 2.2	14.1 ± 2.1	14.5 ± 2.2	14.5 ± 2.2	< 0.001
Triglyceride (mg/dL)	129.7 ± 96.2	117.7 ± 83.9	127.0 ± 87.3	141.5 ± 108.2	< 0.001
Total cholesterol (mg/dL)	193.9 ± 37.5	189.6 ± 35.4	192.5 ± 37.5	198.4 ± 38.7	< 0.001
LDL-C (mg/dL)	117.0 ± 34.3	114.1 ± 32.1	116.1 ± 33.8	120.0 ± 36.0	< 0.001
HDL-C (mg/dL)	51.7 ± 15.3	52.9 ± 15.0	51.6 ± 15.8	50.7 ± 15.3	< 0.001
eGFR (ml/min/1.72m^2^)	96.7 ± 19.4	98.9 ± 19.3	97.3 ± 18.9	94.4 ± 19.6	< 0.001
FBG (mg/dL)	109.0 ± 34.2	104.2 ± 25.5	108.5 ± 32.1	113.4 ± 40.5	< 0.001
HbA1c (%)	5.2 ± 0.8	5.2 ± 0.6	5.3 ± 0.8	5.3 ± 0.9	< 0.001
Uric acid (mg/dL)	4.4 ± 1.2	4.2 ± 1.1	4.5 ± 1.2	4.6 ± 1.3	< 0.001
C-reactive protein (mg/l)*	1.0 (0.5, 2.0)	0.8 (0.5, 1.7)	0.9 (0.5, 2.0)	1.1 (0.6, 2.3)	< 0.001
TyG	8.7 ± 0.7	8.5 ± 0.6	8.6 ± 0.7	8.8 ± 0.7	< 0.001
**Self reported comorbidity**					
Current smoker, n(%)	1985 (30.3)	697 (28.6)	428 (32.3)	860 (30.7)	0.050
Current drinker, n(%)	2235 (34.1)	813 (33.4)	477 (36.0)	945 (33.8)	0.238
Hypertension, n(%)	1329 (20.3)	160 (6.6)	193 (14.6)	976 (34.9)	< 0.001
Anti-hypertensive treatment, n(%)	1001 (15.3)	109 (4.5)	145 (11.0)	747 (26.7)	< 0.001
Diabetes mellitus, n(%)	289 (4.4)	82 (3.4)	58 (4.4)	149 (5.3)	0.003
Anti-diabetic treatment, n(%)	187 (2.9)	46 (1.9)	43 (3.3)	98 (3.5)	0.001
Dyslipidemia, n(%)	471 (7.2)	137 (5.6)	89 (6.7)	245 (8.8)	< 0.001
Lipid-lowering treatment, n(%)	247 (3.8)	72 (3.0)	37 (2.8)	138 (4.9)	< 0.001
CKD, n(%)	396 (6.0)	155 (6.4)	90 (6.8)	151 (5.4)	0.145
Chronic lung disease, n(%)	679 (10.4)	242 (9.9)	143 (10.8)	294 (10.5)	0.673

SBP, systolic blood pressure; DBP, diastolic blood pressure; LDL-C, low density lipoprotein cholesterol; HDL-C, high density lipoprotein cholesterol; eGFR, estimated glomerular filtration rate; FBG, fasting blood glucose; HbA1c, hemoglobin A1c; TyG, triglyceride-glucose index; CKD, chronic kidney disease

* Present as median (interquartile range)

The baseline characteristics of participants stratified by quartile of the TyG index and individuals with and without incident CVD are also presented in Supplemental Tables 1 and 2. In brief, participants with a higher TyG index were less likely to be male, had higher levels of BP and anthropometrics, and had a higher prevalence of hypertension. Compared to those without incident CVD, individuals with CVD were older, comprised a higher proportion of women, and had higher levels of BP, anthropometrics, lipid profiles, and glucose. Furthermore, they tended to have a heavier burden of comorbidities than their counterparts without CVD. (Supplemental Tables 1 and 2)

### Associations between TyG index and CVD among participants with varying SBP statuses

After 58,333 person-years of follow-up, 1599 cases (incidence rate: 27.4 (26.1, 28.8) per 1000 person-years) of CVD were captured, including 1242 cases (incidence rate: 23.0 (21.8, 24.4) per 1000 person-years) of heart disease and 625 cases (incidence rate: 11.0 (10.2, 11.9) per 1000 person-years) of stroke. Among the overall participants, per 1-SD higher TyG index was associated with a 19% (HR: 1.19; 95% CI: 1.12, 1.27) higher risk for incident CVD, and the participants with the highest quartile of TyG index had a 54% (HR: 1.54; 95% CI: 1.29, 1.84) higher risk of CVD compared to those with the lowest quartile. Notably, SBP significantly modifies the association between the TyG index and CVD. When TyG index was considered as a continuous variable, a 1-SD higher TyG index was associated with a 31% (HR: 1.31; 95% CI: 1.17, 1.47), 28% (HR: 1.28; 95% CI: 1.12, 1.47), and 10% (HR: 1.10; 95% CI: 1.01, 1.20) higher risks of CVD among participants with SBP < 120mmHg, SBP between 120 and 129mmHg, and SBP ≥ 130mmHg, respectively. The relative HR with an increment in the SBP group was 0.91 (95% CI: 0.86, 0.96) (p-interaction < 0.001). When the TyG index was considered as a categorical variable, individuals with Q4 of the TyG index had a 109% (HR: 2.09; 95% CI: 1.53, 2.87), 62% (HR: 1.62; 95% CI: 1.09, 2.41), and 23% (HR: 1.23; 95% CI: 0.95, 1.60) higher risk for CVD compared to their counterparts in Q1 of the TyG index (p for interaction < 0.001). (Table 2) All the covariates included in the multi-adjusted model had VIF values < 5. (Supplemental Table 3)

**Table 2:** Associations between triglyceride-glucose index and incident cardiovascular disease stratified by systolic blood pressure

TyG	Overall (*N* = 6558)		SBP < 120mmHg (*N* = 2434)		SBP = 120-129mmHg (*N* = 1324)		SBP ≥ 130mmHg (*N* = 2800)		Relative HR with SBP group increment	P-interaction
	Incidence rate & 95% CI (Per 1000 person-years)	HR (95% CI)	Incidence rate & 95% CI (Per 1000 person-years)	HR (95% CI)	Incidence rate & 95% CI (Per 1000 person-years)	HR (95% CI)	Incidence rate & 95% CI (Per 1000 person-years)	HR (95% CI)		
Per 1-SD increment	27.4 (26.1, 28.8)	**1.19 (1.12, 1.27)**	21.8 (19.9, 23.9)	**1.31 (1.17, 1.47)**	28.3 (25.4, 31.5)	**1.28 (1.12, 1.47)**	31.9 (29.7, 34.2)	**1.10 (1.01, 1.20)**	**0.91 (0.86, 0.96)**	**< 0.001**
Q1[< 8.21]	17.3 (15.3, 19.6)	Reference	13.4 (10.9, 16.5)	Reference	17.9 (13.8, 23.3)	Reference	22.4 (18.6, 27.1)	Reference	Reference	**< 0.001**
Q2[8.21 ∼ 8.58]	24.7 (22.2, 27.3)	**1.29 (1.10, 1.52)**	18.9 (15.7, 22.7)	1.32 (0.99, 1.75)	25.8 (20.6, 32.3)	1.32 (0.92, 1.88)	30.1 (25.9, 35.0)	1.23 (0.96, 1.58)	0.96 (0.88, 1.04)	
Q3[8.58 ∼ 9.02]	30.7 (28.0, 33.7)	**1.48 (1.25, 1.74)**	25.4 (21.4, 30.3)	**1.63 (1.22, 2.18)**	32.7 (26.7, 40.1)	**1.63 (1.14, 2.32)**	33.7 (29.6, 38.3)	**1.28 (1.00, 1.63)**	**0.87 (0.77, 0.98)**	
Q4[> 9.02]	37.1 (34.1, 40.4)	**1.54 (1.29, 1.84)**	35.5 (30.2, 41.7)	**2.09 (1.53, 2.87)**	37.9 (31.4, 45.7)	**1.62 (1.09, 2.41)**	37.7 (33.6, 42.4)	1.23 (0.95, 1.60)	**0.76 (0.65, 0.89)**	
P-trend	-	**< 0.001**	-	**< 0.001**	-	**0.010**	-	0.184	**-**	**-**

TyG, triglyceride-glucose index; SBP, systolic blood pressure; HR, hazard ratio; CI, confidence interval; SD, standard deviation

The **bold font** indicates significance

**Fig. 1:**
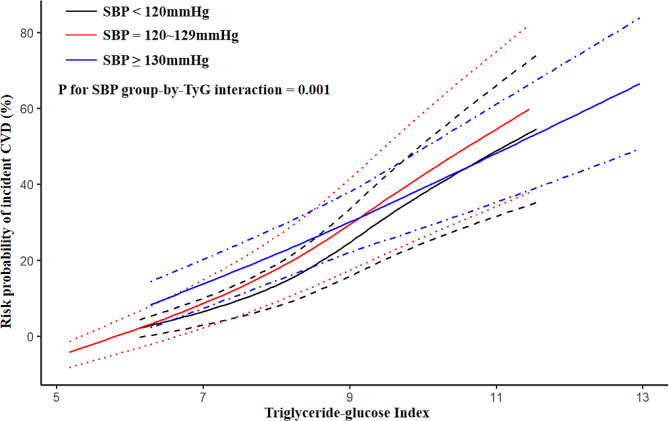
Associations between triglyceride-glucose index and risk probability of incident cardiovascular disease stratified by systolic blood pressure SBP: systolic blood pressure; TyG: triglyceride-glucose index; CVD: cardiovascular disease

**Fig. 2:**
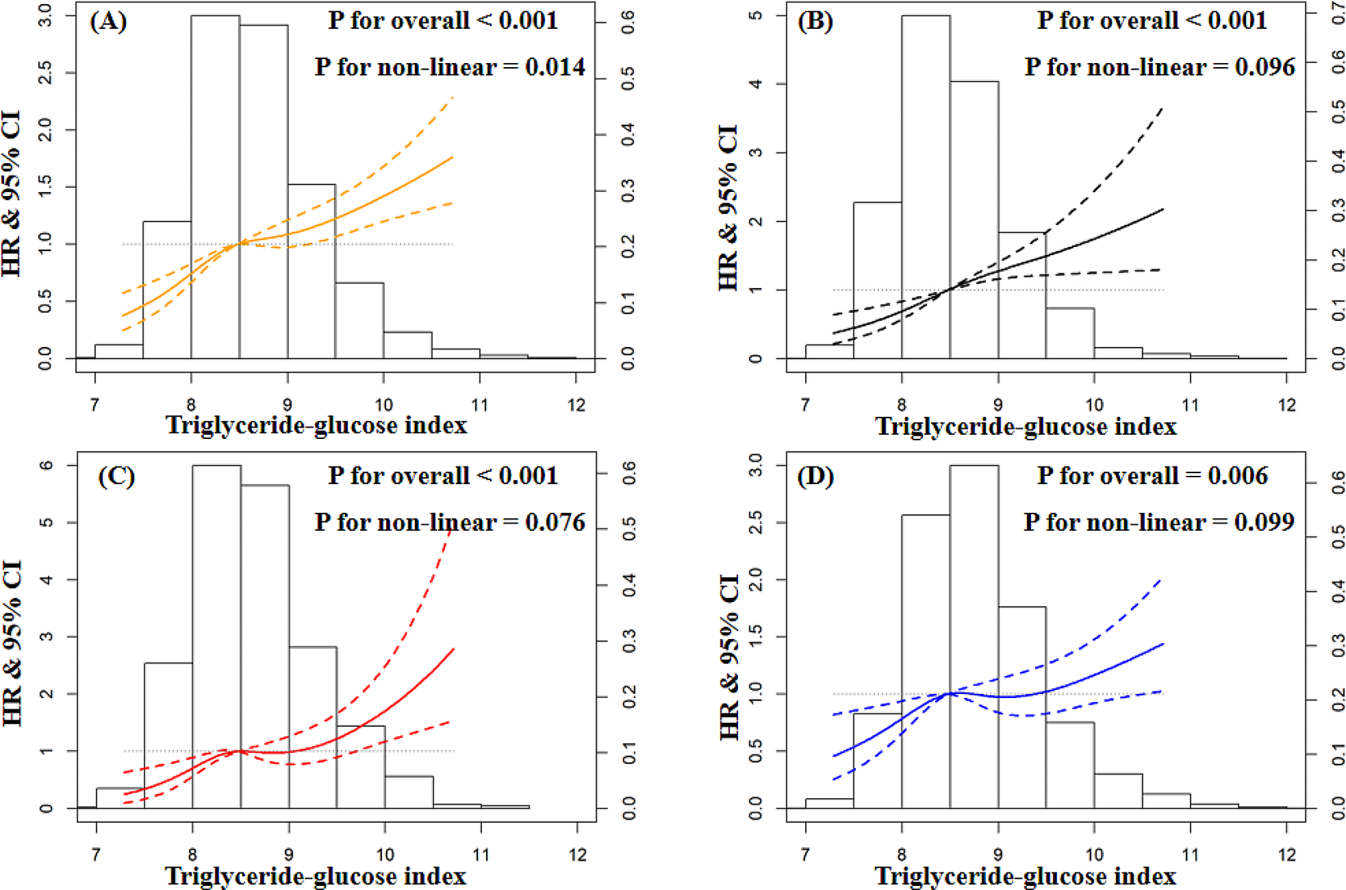
Restricted cubic spline analysis of triglyceride-glucose index with incident cardiovascular disease stratified by systolic blood pressure Panel **A**: Non-linear relationship between TyG and incident CVD among overall participants Panel **B**: Non-linear relationship between TyG and incident CVD among participants with SBP < 120mmHg Panel **C**: Non-linear relationship between TyG and incident CVD among participants with SBP between 120 and 129mmHg Panel **D**: Non-linear relationship between TyG and incident CVD among participants with SBP ≥ 130mmHg HR: hazard ratio; CI: confidence interval

As shown in Fig. 1, at a low level of TyG index, individuals with SBP ≥ 130mmHg had the highest risk probability of CVD, followed by those with SBP between 120 and 129mmHg and SBP < 120mmHg. With a higher TyG index, the risk increased more steeply among participants with SBP less than 130mmHg and exceeded those with the highest SBP level (p for interaction = 0.001). (Fig. 1) The RCS analyses revealed a linear relationship between the TyG index and CVD among participants with varying SBP statuses, with the HR increasing slowly at high levels of the TyG index among participants with high SBP. (Fig. 2)

### Mediator analysis

**Fig. 3:**
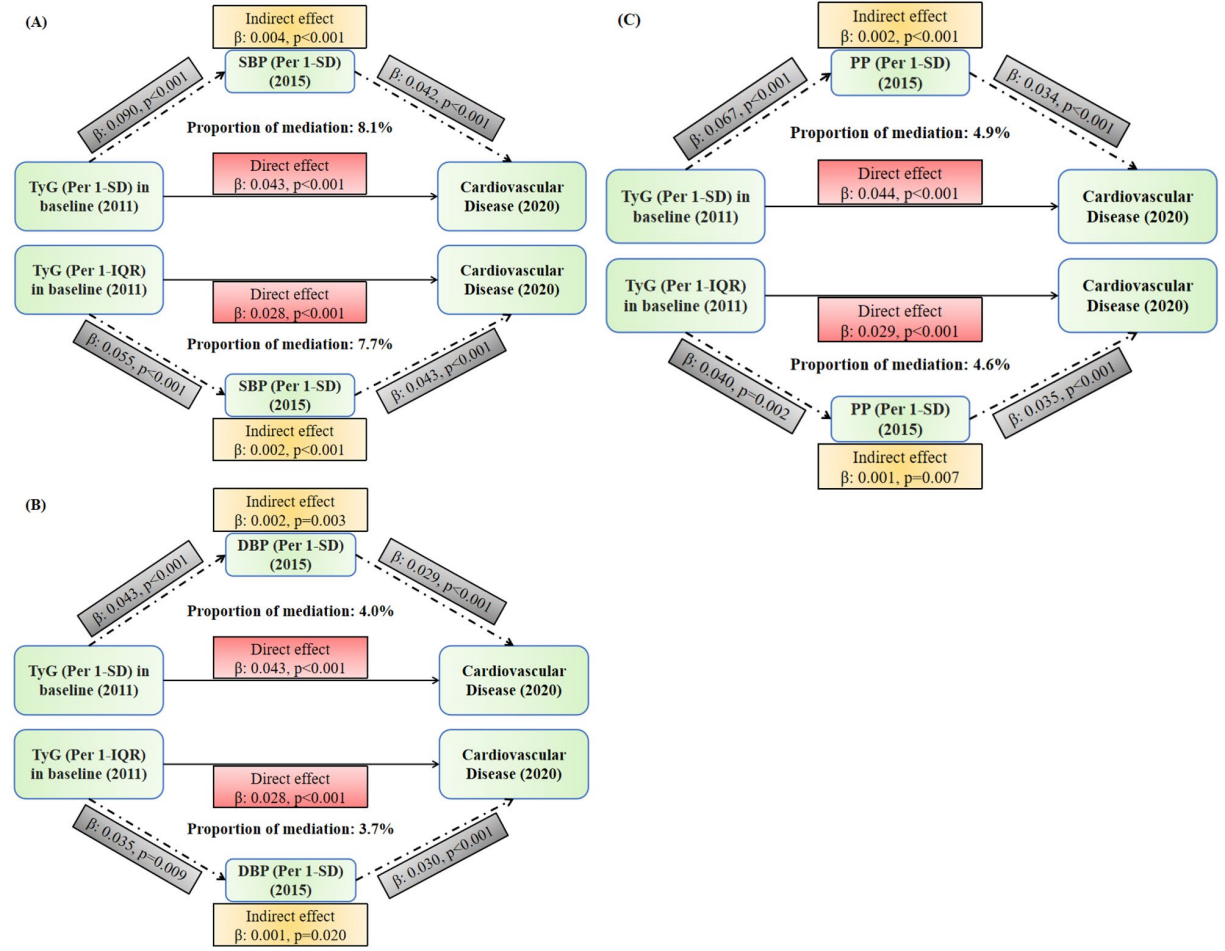
Mediated effects of blood pressure on the association between the triglyceride-glucose index with incident cardiovascular disease TyG: triglyceride-glucose index; SBP: systolic blood pressure; DBP: diastolic blood pressure; PP: pulse pressure; SD: standard deviation: IQR: interquartile range

The mediator analyses indicated that significant indirect effects of SBP existed between the TyG index and incident CVD, with mediation at approximately 8.1% (per 1-SD of TyG index) and 7.7% (per 1-IQR of TyG index) of the associations with CVD (Fig. 3 Panel A). In contrast, DBP and PP had a relatively lower proportion of mediation between TyG and incident CVD, mediating around 3.7–4.9% of the associations. (Fig. 3 Panels B and C) Regarding the individual CVD components, SBP significantly mediated over 10% of the associations between TyG and the risks of incident heart disease and stroke. In contrast, lower or even non-significant mediated effects of DBP and PP on the associations between TyG with heart disease and stroke were observed. (Supplemental Figs. 2–3)

### Additional and sensitivity analysis

Among the secondary outcomes, the TyG index was independently associated with higher HRs for incident heart disease and stroke among all participants. Additionally, significant modified effects of SBP on the associations between the TyG index with heart disease and stroke were observed (Supplemental Tables 4 and 5, Supplemental Figs. 4–6).

When participants were categorized based on their DBP levels, the significant modified effect of DBP status was only observed on the associations between the TyG index and incident heart disease (Supplemental Tables 6–8, Supplemental Figs. 8–10). When participants were categorized based on their PP levels, significant modified effects of PP status were observed on the associations between the TyG index with incident CVD and stroke. No modified effect was observed on the relationship between the TyG index and heart disease (Supplemental Tables 9–11, Supplemental Figs. 11–14).

After excluding participants with self-reported DM, with HbA1c or FBG ≥ 126 mg/dL, the observed results remained consistent. (Supplemental Table 12)

## Discussion

Our study has the following novel findings. First, SBP status significantly modified the associations between the TyG index and incident CVD, with greater HRs for CVD observed among individuals with optimal and normal SBP (less than 130mmHg). The conclusions remained valid in the analysis of the associations between the TyG index with heart disease and stroke. Furthermore, a similar modified effect on the TyG index and CVD was also observed in the PP. Lastly, SBP mediated a significant proportion of the associations between the TyG index with incident CVD.

In line with previous studies [23–25], our study re-emphasized that the TyG index was an independent predictor for incident CVD among general middle-aged and elderly populations. Moreover, several recent studies have demonstrated that common risk factors could intertwine and modify their effect on the relationship between the TyG index and CVD [13, 26, 27]. In another study conducted within the CHARLS cohort, Huo et al. discovered significant joint and interacting effects of the TyG index and BMI on incident stroke among 8231 Chinese adults, with the TyG index found to mediate more than 50% of the association between BMI and CVD [13]. On the contrary, in the Kailuan cohort, Tian et al. showed that the total association between obesity and CVD was reversely mediated by the TyG index by over 30% [27]. Furthermore, the TyG index could effectively enhance the discrimination and reclassification of traditional risk factors for CVD [28]. However, there has been no study to comprehensively clarify the intertwined effects of BP and the TyG index on incident CVD to date. We for the first time found that SBP status significantly modified the associations between the TyG index and CVD, with greater risks of CVD observed among participants with optimal and normal SBP levels. The conclusion remained consistent when analyzing the associations between the TyG index with CVD among subjects with varying PP statuses. Additionally, with a higher TyG index, the estimated risk probability of CVD among participants with SBP less than 130mmHg even surpassed those with hypertension. However, contradicting the current study, a recent Kailuan Study showed that simultaneous elevations of TyG and BP had a joint effect on CVD risks [16]. The potential reason for the discrepancy might be the cumulative TyG index and BP employed in the Kailuan Study over 5 years [16], and those with both high cumulative TyG and BP in this study may represent an unhealthy sample due to long-term exposure. Nevertheless, as an observational study conducted among Chinese middle-aged and elderly individuals, the conclusions need to be further confirmed.

There were several possible explanations for the underlying mechanisms of the varying degrees in the relationships between the TyG index and CVD among participants with different BP statuses. First, high SBP is the primary risk factor for CVD [1, 2]. In other words, participants with high SBP might represent a selected population in whom “BP takes it all”, which masks or even conceals the impacts of other cardio-metabolic risk factors. What’s more, a significant mediation effect of SBP on the associations between the TyG index with CVD was observed in both our study and a prior one [15], This implies that BP may act as a link between them, indicating that the adverse effects of the TyG index could partly lead to elevated BP, thereby accelerating the development of CVD. Indeed, prior studies have demonstrated a positive relationship between the TyG index and incident hypertension [29–33]. What’s worse is that nearly two-thirds (65.1%) of the participants were unaware of their hypertensive condition (≥ 130mmHg) and only approximately 15% of participants had the SBP at the target level (< 130mmHg) in the current study (Table 1). These potential risk factors may exacerbate the hazardous effect of hypertension and diminish the impact of the TyG index since both intensive BP treatment [34] and increased awareness of hypertension [35, 36] bring benefits to the elderly population. Nevertheless, due to the observational nature of the present study, the potential mechanisms behind the observations warrant further investigation.

An integrated analysis of the varying impacts of the TyG index on CVD across different BP statuses is crucial for precise prevention and enhancing shared decision-making. First, insulin resistance was a significant predictor of future CVD among participants with varying BP statuses, especially in those with ideal BP. Nonetheless, undermanagement of cardiovascular risk factors is alarmingly common in China, and it is reported that an extremely limited number of Chinese people maintain optimal cardiovascular health [37, 38]. As demonstrated in the current study, both the awareness of high SBP and the percentage of participants with normal SBP remained low among Chinese middle-aged and elderly individuals. Our study emphasizes the importance of increasing awareness of hypertension and enhancing BP management as a priority for CVD prevention. When the BP is within the normal range, people need to pay closer attention to other risk factors (e.g., the TyG index) management. Second, we found that SBP partially mediated the effects of the TyG index on incident CVD, highlighting that the TyG index also has a detrimental impact on BP progression. This underscores the importance of early identification of individuals with increasing TyG index and timely intervention as an effective and efficient strategy for preventing hypertension. Third, the TyG index was strongly associated with CVD in participants with different PP statuses, and this association was partially mediated by PP. Although DBP does not have comparable impacts as SBP on health [39], PP, a well-recognized surrogate of arterial stiffness calculated by subtracting DBP from SBP, is a critical predictor for CVD [40]. In line with previous studies [41, 42], our study emphasized that the TyG index was an independent risk factor for PP -- a representation of arterial stiffness, and provided insights for the prevention of atherosclerosis. Moreover, we emphasized the importance of screening for the TyG index to prevent future CVD, especially before atherosclerosis develops.

There were several noteworthy limitations in the current study. First, we categorized the participants based solely on their baseline BP levels and quartile of the TyG index without considering their temporal changes when assessing their associations with CVD. Numerous studies have found that variations and trends in BP and TyG are useful for identifying individuals at high CVD risk [29, 43]. Furthermore, categorizations based on a single measurement of BP and TyG might lead to misclassifications. Second, as an observational study, inherent biases, such as residual confounding and reliable causality, cannot be avoided and established. Third, this study was conducted among Chinese middle-aged and elderly adults; therefore, the generalizability of our conclusions to other races or younger age groups should be limited and needs further confirmation. Fourth, baseline information on demographics and comorbidities was self-reported, which may lead to recall bias and inaccurate estimation of comorbid prevalence. Last but not least, the study outcomes were collected through personal interviews with participants without formal adjudication. Future studies using independently adjudicated endpoints are warranted to confirm these results.

## Conclusions

Our study found that the associations between the TyG index and incident CVD were modified by BP status, with greater HRs for CVD observed among Chinese middle-aged and elderly adults with SBP < 130mmHg. Furthermore, SBP can partially mediate the association between the TyG index with CVD. The present study highlights the importance of early screening for the TyG index to identify individuals at risk of hypertension and CVD.

### Electronic supplementary material

Below is the link to the electronic supplementary material.

Supplementary Material 1

## Data Availability

The data analyzed in this study are available from the Institute of Social Science Survey, Peking University, Beijing, China. (http://charls.pku.edu.cn).
