# Supplementary material for: Systolic blood pressure status modifies the associations between the triglyceride-glucose index and incident cardiovascular disease: a national cohort study in China
Source: Cardiovasc Diabetol. 2024 Apr 24;23:135. doi: 10.1186/s12933-024-02227-w (PMC11044345; doi:10.1186/s12933-024-02227-w)
Supplement: Supplementary file 1 — Supplementary Material 1 [file 12933_2024_2227_MOESM1_ESM.docx]

**Supplemental materials**

**Supplemental Table 1. Baseline characteristics comparison stratified by the quartile of TyG.**

| **Variables** | **Quartile of TyG** | | | | **P-value** |
| --- | --- | --- | --- | --- | --- |
|  | **Q [<8.21]**  **(N=1640)** | **Q2 [8.21~8.58] (N=1640)** | **Q3 [8.58~9.02] (N=1639)** | **Q4 [>9.02] (N=1639)** |  |
| **Demographic** | | | | | |
| Age (years) | 58.2±9.1 | 58.3±8.7 | 58.8±8.7 | 58.1±8.4 | 0.073 |
| Male, n(%) | 886 (54.0) | 768 (46.8) | 671 (40.9) | 692 (42.2) | <0.001 |
| Married, n(%) | 1453 (88.6) | 1472 (89.8) | 1475 (90.0) | 1484 (90.5) | <0.001 |
| Rural residence, n(%) | 1397 (85.2) | 1377 (84.0) | 1358 (82.9) | 1332 (81.3) | 0.049 |
| Educational attainment < High school, n(%) | 1481 (90.3) | 1470 (89.6) | 1488 (90.8) | 1480 (90.3) | <0.001 |
| **Physical examination** | | | | | |
| SBP (mmHg) | 125.1±20.2 | 127.5±20.0 | 131.1±21.5 | 133.2±20.9 | <0.001 |
| DBP (mmHg) | 73.2±11.9 | 74.6±11.8 | 76.1±11.8 | 77.7±11.8 | <0.001 |
| Pulse (beat per minute) | 70.5±9.8 | 71.5±9.6 | 72.5±10.2 | 73.7±10.3 | <0.001 |
| Body mass index (kg/m^2^) | 22.1±3.3 | 22.9±3.9 | 23.8±3.9 | 24.9±3.8 | <0.001 |
| Waist circumference (cm) | 80.0±11.4 | 82.6±11.5 | 85.0±12.5 | 88.2±12.3 | <0.001 |
| **Laboratory** | | | | | |
| Hemoglobin (g/dL) | 14.2±2.3 | 14.3±2.2 | 14.3±2.2 | 14.6±2.1 | <0.001 |
| Triglyceride (mg/dL) | 60.3±13.4 | 90.0±14.6 | 126.7±22.2 | 241.9±131.1 | <0.001 |
| Total cholesterol (mg/dL) | 180.3±32.5 | 190.5±33.9 | 197.0±36.7 | 208.0±40.8 | <0.001 |
| LDL-C (mg/dL) | 109.4±28.4 | 119.3±30.9 | 123.9±34.0 | 115.4±40.8 | <0.001 |
| HDL-C (mg/dL) | 60.9±15.2 | 55.1±13.9 | 49.4±13.1 | 41.4±11.7 | <0.001 |
| eGFR (ml/min/1.72m^2^) | 99.6±19.2 | 97.9±19.1 | 95.1±19.3 | 94.1±19.7 | <0.001 |
| FBG (mg/dL) | 99.5±14.0 | 101.2±14.3 | 106.3±18.1 | 133.0±55.8 | <0.001 |
| HbA1c (%) | 5.1±0.4 | 5.1±0.5 | 5.2±0.5 | 5.6±1.2 | <0.001 |
| Uric acid (mg/dL) | 4.3±1.1 | 4.3±1.2 | 4.5±1.2 | 4.7±1.3 | <0.001 |
| C-reactive protein (mg/l)* | 0.8 (0.4, 1.7) | 0.9 (0.5, 1.8) | 1.0 (0.6, 2.0) | 1.3 (0.7, 2.5) | <0.001 |
| **Self-reported comorbidity** | | | | | |
| Current smoker, n(%) | 569 (34.7) | 532 (32.4) | 442 (27.0) | 442 (27.0) | <0.001 |
| Current drinker, n(%) | 642 (39.2) | 579 (35.3) | 479 (29.2) | 535 (32.6) | <0.001 |
| Hypertension, n(%) | 206 (12.6) | 266 (16.2) | 369 (22.5) | 488 (29.8) | <0.001 |
| Anti-hypertensive treatment, n(%) | 147 (9.0) | 182 (11.1) | 286 (17.5) | 386 (23.6) | <0.001 |
| Diabetes mellitus, n(%) | 26 (1.6) | 52 (3.2) | 58 (3.5) | 153 (9.3) | <0.001 |
| Anti-diabetic treatment, n(%) | 16 (1.0) | 27 (1.7) | 30 (1.8) | 114 (7.0) | <0.001 |
| Dyslipidemia, n(%) | 57 (3.5) | 100 (6.1) | 115 (7.0) | 199 (12.1) | <0.001 |
| Lipid-lowering treatment, n(%) | 25 (1.5) | 47 (2.9) | 64 (3.9) | 111 (6.8) | <0.001 |
| CKD, n(%) | 104 (7.3) | 115 (7.0) | 95 (5.8) | 82 (5.0) | 0.099 |
| Chronic lung disease, n(%) | 191 (11.7) | 173 (10.6) | 153 (9.3) | 162 (9.9) | 0.155 |

TyG, triglyceride-glucose index; SBP, systolic blood pressure; DBP, diastolic blood pressure; LDL-C, low density lipoprotein cholesterol; HDL-C, high density lipoprotein cholesterol; eGFR, estimated glomerular filtration rate; FBG, fasting blood glucose; HbA1c, hemoglobin A1c; CKD, chronic kidney disease.

* Present as median (interquartile range), and log transfHRmed when being tested in the linear regression model.

**Supplemental Table 2. Baseline characteristics comparison among participants with and without incident cardiovascular disease.**

| **Variables** | **Without incident CVD (N=4959)** | **With incident CVD (N=1599)** | **P-value** |
| --- | --- | --- | --- |
| **Demographic** | | |  |
| Age (years) | 58.1±8.9 | 59.1±8.1 | <0.001 |
| Male, n(%) | 2362 (47.6) | 655 (41.0) | <0.001 |
| Married, n(%) | 4447 (89.7) | 1437 (89.9) | 0.825 |
| Rural residence, n(%) | 4154 (83.8) | 1310 (81.9) | 0.086 |
| Educational attainment < High school, n(%) | 4482 (90.4) | 1437 (89.9) | 0.548 |
| **Physical examination** | | |  |
| SBP (mmHg) | 128.0±20.6 | 132.8±21.4 | <0.001 |
| DBP (mmHg) | 73.2±11.9 | 74.6±11.8 | <0.001 |
| Pulse (beat per minute) | 74.8±11.8 | 77.3±12.1 | <0.001 |
| Body mass index (kg/m^2^) | 23.2±3.7 | 24.4±4.3 | <0.001 |
| Waist circumference (cm) | 83.1±11.9 | 86.5±13.2 | <0.001 |
| **Laboratory** | | |  |
| Hemoglobin (g/dL) | 14.3±2.2 | 14.5±2.2 | 0.013 |
| Triglyceride (mg/dL) | 122.5±88.4 | 152.3±114.4 | <0.001 |
| Total cholesterol (mg/dL) | 192.3±37.2 | 198.9±38.0 | <0.001 |
| LDL-C (mg/dL) | 116.2±33.6 | 119.4±36.2 | 0.001 |
| HDL-C (mg/dL) | 52.5±15.4 | 49.2±14.9 | <0.001 |
| eGFR (ml/min/1.72m^2^) | 97.2±19.6 | 95.1±19.0 | <0.001 |
| FBG (mg/dL) | 107.2±31.2 | 114.7±41.5 | <0.001 |
| HbA1c (%) | 5.2±0.7 | 5.4±1.0 | <0.001 |
| Uric acid (mg/dL) | 4.4±1.2 | 4.5±1.3 | 0.428 |
| C-reactive protein (mg/l)* | 0.9 (0.5, 1.9) | 1.2 (0.6, 2.3) | <0.001 |
| TyG | 8.6±0.6 | 8.9±0.7 | <0.001 |
| **Self-reported comorbidity** | | |  |
| Current smoker, n(%) | 1558 (31.4) | 427 (26.7) | <0.001 |
| Current drinker, n(%) | 1732 (34.9) | 503 (31.5) | 0.011 |
| Hypertension, n(%) | 838 (16.9) | 491 (30.7) | <0.001 |
| Anti-hypertensive treatment, n(%) | 618 (12.5) | 383 (24.0) | <0.001 |
| Diabetes mellitus, n(%) | 173 (3.5) | 116 (7.3) | <0.001 |
| Anti-diabetic treatment, n(%) | 110 (2.2) | 77 (4.8) | <0.001 |
| Dyslipidemia, n(%) | 264 (5.3) | 207 (13.0) | <0.001 |
| Lipid-lowering treatment, n(%) | 127 (2.6) | 120 (7.5) | <0.001 |
| CKD, n(%) | 280 (5.7) | 116 (7.3) | 0.019 |
| Chronic lung disease, n(%) | 452 (9.1) | 227 (14.2) | <0.001 |

CVD, cardiovascular disease; SBP, systolic blood pressure; DBP, diastolic blood pressure; LDL-C, low density lipoprotein cholesterol; HDL-C, high density lipoprotein cholesterol; eGFR, estimated glomerular filtration rate; FBG, fasting blood glucose; HbA1c, hemoglobin A1c; TyG, triglyceride-glucose index; CKD, chronic kidney disease.

* Present as median (interquartile range), and compare using Mann-Whitney test.

**Supplemental Table 3. Variance inflation factor of the adjusted covariates.**

| **Covariate** | **VIF** |
| --- | --- |
| TyG | 1.49 |
| Age | 1.32 |
| Sex | 2.56 |
| Marital status | 1.10 |
| Educational attainment | 1.09 |
| Residential area | 1.07 |
| SBP | 1.26 |
| pulse | 1.04 |
| BMI | 1.75 |
| Waist circumference | 1.57 |
| Hemoglobin | 1.22 |
| LDL-C | 1.08 |
| HDL-C | 1.46 |
| Uric acid | 1.54 |
| eGFR | 1.32 |
| C-reactive protein | 1.02 |
| Hemoglobin A1c | 1.30 |
| Smoking status | 1.96 |
| Drinking status | 1.41 |
| Hypertension | 2.86 |
| Anti-hypertensive treatment | 2.83 |
| Diabetes mellitus | 2.84 |
| Anti-diabetic treatment | 2.92 |
| Dyslipidemia | 2.12 |
| Lipid-lowering treatment | 2.09 |
| Chronic lung disease | 1.02 |
| Mean VIF | 1.66 |

VIF, variance inflation factor; TyG, triglyceride-glucose index; SBP, systolic blood pressure; BMI, body mass index; LDL-C, low density lipoprotein cholesterol; HDL-C, high density lipoprotein cholesterol; eGFR, estimated glomerular filtration rate; CKD, chronic kidney disease.

**Supplemental Table 4. Associations between triglyceride-glucose index and incident heart disease stratified by systolic blood pressure.**

| **TyG** | **Overall (N=6558)** | | **SBP < 120mmHg (N=2434)** | | **SBP = 120-129mmHg (N=1324)** | | **SBP ≥ 130mmHg (N=2800)** | | **Relative HR with SBP group increment** | **P-interaction** |
| --- | --- | --- | --- | --- | --- | --- | --- | --- | --- | --- |
|  | **Incidence rate & 95% CI (Per 1000 person-years)** | **HR (95% CI)** | **Incidence rate & 95% CI (Per 1000 person-years)** | **HR (95% CI)** | **Incidence rate & 95% CI (Per 1000 person-years)** | **HR (95% CI)** | **Incidence rate & 95% CI (Per 1000 person-years)** | **HR (95% CI)** |  |  |
| Per 1-SD increment | 23.0 (21.8, 24.4) | **1.10 (1.02, 1.18)** | 18.0 (16.2, 19.9) | **1.22 (1.07, 1.40)** | 24.4 (21.6, 27.5) | 1.09 (0.93, 1.27) | 27.0 (24.9, 29.2) | 1.04 (0.95, 1.15) | **0.90 (0.85, 0.96）** | **0.002** |
| Q1[<8.21] | 16.6 (14.6, 18.9) | Reference | 13.0 (10.5, 16.1) | Reference | 19.1 (14.7, 24.9) | Reference | 20.0 (16.3, 24.7) | Reference | Reference | **0.001** |
| Q2[8.21~8.58] | 21.7 (19.3, 24.3) | 1.19 (0.99, 1.41) | 14.9 (12.0, 18.5) | 1.10 (0.80, 1.49) | 23.3 (18.2, 29.8) | 1.08 (0.74, 1.57) | 28.2 (24.0, 33.2) | 1.30 (0.99, 1.70) | 0.95 (0.87, 1.05) |  |
| Q3[8.58~9.02] | 24.3 (21.8, 27.1) | **1.23 (1.03, 1.48)** | 19.3 (15.7, 23.7) | 1.30 (0.94, 1.79) | 24.9 (19.5, 31.7) | 1.10 (0.74, 1.62) | 28.0 (24.1, 32.5) | 1.23 (0.94, 1.62) | **0.85 (0.75, 0.97)** |  |
| Q4[>9.02] | 29.9 (27.1, 33.0) | **1.34 (1.10, 1.63)** | 29.6 (24.6, 35.7) | **1.86 (1.32, 2.63)** | 30.9 (24.9, 38.5) | 1.12 (0.73, 1.73) | 29.7 (25.9, 34.1) | 1.20 (0.89, 1.61) | **0.77 (0.64, 0.92)** |  |
| P-trend | - | **0.005** | - | **<0.001** | - | 0.610 | - | 0.433 | **-** | **-** |

TyG, triglyceride-glucose index; SBP, systolic blood pressure; HR, hazard ratio; CI, confidence interval; SD, standard deviation.

The **bold font** indicates significance.

**Supplemental Table 5. Associations between triglyceride-glucose index and incident stroke stratified by systolic blood pressure.**

| **TyG** | **Overall (N=6558)** | | **SBP < 120mmHg (N=2434)** | | **SBP = 120-129mmHg (N=1324)** | | **SBP ≥ 130mmHg (N=2800)** | | **Relative HR with SBP group increment** | **P-interaction** |
| --- | --- | --- | --- | --- | --- | --- | --- | --- | --- | --- |
|  | **Incidence rate & 95% CI (Per 1000 person-years)** | **HR (95% CI)** | **Incidence rate & 95% CI (Per 1000 person-years)** | **HR (95% CI)** | **Incidence rate & 95% CI (Per 1000 person-years)** | **HR (95% CI)** | **Incidence rate & 95% CI (Per 1000 person-years)** | **HR (95% CI)** |  |  |
| Per 1-SD increment | 11.0 (10.2, 11.9) | **1.22 (1.11, 1.34)** | 8.0 (6.8, 9.3) | **1.39 (1.15, 1.68)** | 9.7 (8.0, 11.7) | **1.54 (1.22, 1.95)** | 14.4 (13.0, 16.1) | 1.09 (0.96, 1.24) | **0.91 (0.83, 0.99）** | **0.022** |
| Q1[<8.21] | 5.9 (4.8, 7.3) | Reference | 3.9 (2.7, 5.8) | Reference | 3.6 (2.0, 6.5) | Reference | 10.3 (7.8, 13.7) | Reference | Reference | **0.001** |
| Q2[8.21~8.58] | 9.6 (8.1, 11.3) | **1.42 (1.07, 1.87)** | 7.3 (5.4, 9.8) | **1.71 (1.04, 2.82)** | 7.3 (4.8, 11.2) | **2.14 (1.02, 4.51)** | 13.2 (10.5, 16.7) | 1.14 (0.79, 1.66) | 0.93 (0.81, 1.08) |  |
| Q3[8.58~9.02] | 13.3 (11.5, 15.4) | **1.72 (1.30, 2.26)** | 9.4 (7.0, 12.6) | **2.07 (1.23, 3.46)** | 14.4 (10.5, 19.8) | **4.04 (2.00, 8.17)** | 15.7 (12.9, 19.1) | 1.18 (0.82, 1.71) | 0.84 (0.69, 1.02) |  |
| Q4[>9.02] | 15.6 (13.6, 17.8) | **1.54 (1.14, 2.08)** | 14.1 (10.8, 18.3) | **2.64 (1.52, 4.59)** | 14.3 (10.4, 19.6) | **3.73 (1.71, 8.13)** | 16.9 (14.1, 20.3) | 0.92 (0.62, 1.37) | **0.67 (0.51, 0.87)** |  |
| P-trend | - | **0.005** | - | **0.001** | - | **<0.001** | - | 0.549 | **-** | **-** |

TyG, triglyceride-glucose index; SBP, systolic blood pressure; HR, hazard ratio; CI, confidence interval; SD, standard deviation.

The **bold font** indicates significance.

**Supplemental Table 6. Associations between triglyceride-glucose index and incident cardiovascular disease stratified by diastolic blood pressure.**

| **TyG** | **DBP < 80mmHg (N=4379)** | | **DBP = 80-90mmHg (N=1425)** | | **DBP > 90mmHg (N=754)** | | **Relative HR with DBP group increment** | **P-interaction** |
| --- | --- | --- | --- | --- | --- | --- | --- | --- |
|  | **Incidence rate & 95% CI (Per 1000 person-years)** | **HR (95% CI)** | **Incidence rate & 95% CI (Per 1000 person-years)** | **HR (95% CI)** | **Incidence rate & 95% CI (Per 1000 person-years)** | **HR (95% CI)** |  |  |
| Per 1-SD increment | 24.6 (23.1, 26.2) | **1.23 (1.14, 1.34)** | 32.0 (29.0, 35.3) | **1.13 (1.00, 1.28)** | 35.0 (30.8, 39.8) | **1.16 (1.00, 1.34)** | 0.96 (0.89, 1.04） | 0.154 |
| Q1[<8.21] | 15.1 (13.0, 17.6) | Reference | 63/275 (22.9) | Reference | 26/145 (17.9) | Reference | Reference | 0.146 |
| Q2[8.21~8.58] | 28.5 (25.3, 32.1) | **1.41 (1.15, 1.73)** | 78/344 (22.7) | 0.88 (0.63, 1.23) | 49/165 (29.7) | 1.60 (0.99, 2.61) | 1.01 (0.90, 1.14) |  |
| Q3[8.58~9.02] | 28.5 (25.3, 32.1) | **1.62 (1.32, 2.00)** | 117/388 (30.2) | 1.13 (0.82, 1.57) | 60/190 (31.6) | 1.57 (0.96, 2.56) | 0.97 (0.83, 1.13) |  |
| Q4[>9.02] | 34.3 (30.6, 38.5) | **1.73 (1.38, 2.18)** | 147/418 (35.2) | 1.13 (0.80, 1.60) | 98/254 (38.6) | **1.74 (1.05, 2.88)** | 0.90 (0.74, 1.09) |  |
| P-trend | - | **<0.001** | - | 0.228 | - | 0.070 | **-** | **-** |

TyG, triglyceride-glucose index; DBP, diastolic blood pressure; HR, hazard ratio; CI, confidence interval; SD, standard deviation.

The **bold font** indicates significance.

**Supplemental Table 7. Associations between triglyceride-glucose index and incident heart disease stratified by diastolic blood pressure.**

| **TyG** | **DBP < 80mmHg (N=4379)** | | **DBP = 80-90mmHg (N=1425)** | | **DBP > 90mmHg (N=754)** | | **Relative HR with DBP group increment** | **P-interaction** |
| --- | --- | --- | --- | --- | --- | --- | --- | --- |
|  | **Incidence rate & 95% CI (Per 1000 person-years)** | **HR (95% CI)** | **Incidence rate & 95% CI (Per 1000 person-years)** | **HR (95% CI)** | **Incidence rate & 95% CI (Per 1000 person-years)** | **HR (95% CI)** |  |  |
| Per 1-SD increment | 20.7 (19.3, 22.3) | **1.15 (1.05, 1.26)** | 26.9 (24.1, 30.1) | 1.04 (0.90, 1.20) | 29.5 (25.5, 34.2) | 1.03 (0.86, 1.22) | **0.93 (0.87, 1.00）** | **0.049** |
| Q1[<8.21] | 14.5 (12.4, 17.0) | Reference | 23.2 (17.7, 30.5) | Reference | 21.8 (14.8, 32.0) | Reference | Reference | **0.026** |
| Q2[8.21~8.58] | 19.3 (16.7, 22.4) | 1.23 (0.99, 1.54) | 23.9 (18.8, 30.4) | 0.92 (0.63, 1.33) | 33.7 (25.1, 45.3) | 1.44 (0.87, 2.37) | 0.97 (0.86, 1.10) |  |
| Q3[8.58~9.02] | 22.6 (19.7, 26.0) | **1.34 (1.07, 1.69)** | 28.3 (23.0, 34.9) | 1.10 (0.76, 1.58) | 26.2 (19.2, 35.7) | 0.97 (0.57, 1.66) | 0.86 (0.73, 1.02) |  |
| Q4[>9.02] | 28.6 (25.1, 32.6) | **1.53 (1.19, 1.97)** | 30.7 (25.3, 37.3) | 1.11 (0.75, 1.63) | 33.8 (26.7, 42.9) | 1.14 (0.66, 1.95) | **0.81 (0.65, 1.00)** |  |
| P-trend | - | **0.001** | - | 0.414 | - | 0.905 | **-** | **-** |

TyG, triglyceride-glucose index; DBP, diastolic blood pressure; HR, hazard ratio; CI, confidence interval; SD, standard deviation.

The **bold font** indicates significance.

**Supplemental Table 8. Associations between triglyceride-glucose index and incident stroke stratified by diastolic blood pressure.**

| **TyG** | **DBP < 80mmHg (N=4379)** | | **DBP = 80-90mmHg (N=1425)** | | **DBP > 90mmHg (N=754)** | | **Relative HR with DBP group increment** | **P-interaction** |
| --- | --- | --- | --- | --- | --- | --- | --- | --- |
|  | **Incidence rate & 95% CI (Per 1000 person-years)** | **HR (95% CI)** | **Incidence rate & 95% CI (Per 1000 person-years)** | **HR (95% CI)** | **Incidence rate & 95% CI (Per 1000 person-years)** | **HR (95% CI)** |  |  |
| Per 1-SD increment | 9.4 (8.5, 10.5) | **1.27 (1.12, 1.45)** | 13.5 (11.6, 15.8) | 1.16 (0.96, 1.39) | 15.8 (13.0, 19.3) | 1.17 (0.93, 1.47) | 0.96 (0.88, 1.05） | 0.398 |
| Q1[<8.21] | 4.6 (3.5, 6.0) | Reference | 11.0 (7.5, 16.2) | Reference | 7.9 (4.3, 14.8) | Reference | Reference | 0.237 |
| Q2[8.21~8.58] | 8.8 (7.1, 10.9) | **1.74 (1.21, 2.48)** | 10.2 (7.1, 14.6) | 0.80 (0.47, 1.36) | 13.6 (8.7, 21.3) | 1.58 (0.72, 3.48) | 1.04 (0.86, 1.26) |  |
| Q3[8.58~9.02] | 11.8 (9.8, 14.3) | **2.14 (1.49, 3.07)** | 14.4 (10.8, 19.2) | 1.00 (0.60, 1.65) | 19.5 (13.6, 27.9) | **2.19 (1.02, 4.73)** | 1.04 (0.81, 1.32) |  |
| Q4[>9.02] | 13.9 (11.6, 16.7) | **2.05 (1.37, 3.05)** | 17.2 (13.3, 22.1) | 0.82 (0.48, 1.41) | 19.5 (14.3, 26.6) | 1.80 (0.81, 4.02) | 0.88 (0.64, 1.20) |  |
| P-trend | - | **<0.001** | - | 0.697 | - | 0.168 | **-** | **-** |

TyG, triglyceride-glucose index; DBP, diastolic blood pressure; HR, hazard ratio; CI, confidence interval; SD, standard deviation.

The **bold font** indicates significance.

**Supplemental Table 9. Associations between triglyceride-glucose index and incident cardiovascular disease stratified by pulse pressure.**

| **TyG** | **PP < median (51mmHg) (N=3289)** | | **PP ≥ median (51mmHg) (N=3269)** | | **Relative HR with PP group increment** | **P-interaction** |
| --- | --- | --- | --- | --- | --- | --- |
|  | **Incidence rate & 95% CI (Per 1000 person-years)** | **HR (95% CI)** | **Incidence rate & 95% CI (Per 1000 person-years)** | **HR (95% CI)** |  |  |
| Per 1-SD increment | 24.7 (23.0, 26.6) | **1.30 (1.19, 1.43)** | 30.1 (28.2, 32.2) | **1.10 (1.01, 1.19)** | **0.88 (0.80, 0.97）** | **0.007** |
| Q1[<8.21] | 15.8 (13.3, 18.7) | Reference | 19.3 (16.2, 23.1) | Reference | Reference | **0.011** |
| Q2[8.21~8.58] | 20.9 (17.9, 24.4) | 1.22 (0.97, 1.55) | 28.9 (25.1, 33.2) | **1.33 (1.06, 1.68)** | 0.97 (0.85, 1.12) |  |
| Q3[8.58~9.02] | 27.7 (24.0, 31.9) | **1.48 (1.17, 1.88)** | 33.4 (29.5, 37.8) | **1.44 (1.14, 1.82)** | 0.87 (0.71, 1.06) |  |
| Q4[>9.02] | 37.6 (33.2, 42.7) | **1.83 (1.42, 2.37)** | 36.7 (32.7, 41.1) | **1.32 (1.03, 1.69)** | **0.73 (0.55, 0.97)** |  |
| P-trend | - | **<0.001** | - | 0.055 | **-** | **-** |

TyG, triglyceride-glucose index; PP, pulse pressure; HR, hazard ratio; CI, confidence interval; SD, standard deviation.

The **bold font** indicates significance.

**Supplemental Table 10. Associations between triglyceride-glucose index and incident heart disease stratified by pulse pressure.**

| **TyG** | **PP < median (51mmHg) (N=3289)** | | **PP ≥ median (51mmHg) (N=3269)** | | **Relative HR with PP group increment** | **P-interaction** |
| --- | --- | --- | --- | --- | --- | --- |
|  | **Incidence rate & 95% CI (Per 1000 person-years)** | **HR (95% CI)** | **Incidence rate & 95% CI (Per 1000 person-years)** | **HR (95% CI)** |  |  |
| Per 1-SD increment | 21.0 (19.3, 22.7) | **1.17 (1.06, 1.30)** | 25.2 (23.3, 27.2) | 1.04 (0.94, 1.14) | 0.91 (0.82, 1.01） | 0.085 |
| Q1[<8.21] | 15.6 (13.1, 18.6) | Reference | 17.8 (14.8, 21.6) | Reference | Reference | 0.105 |
| Q2[8.21~8.58] | 18.6 (15.7, 22.0) | 1.11 (0.86, 1.42) | 25.2 (21.6, 29.4) | 1.27 (0.99, 1.63) | 0.98 (0.85, 1.14) |  |
| Q3[8.58~9.02] | 21.7 (18.4, 25.6) | 1.18 (0.91, 1.53) | 26.8 (23.2, 30.9) | 1.28 (0.99, 1.66) | 0.88 (0.70, 1.10) |  |
| Q4[>9.02] | 30.3 (26.2, 35.2) | **1.51 (1.14, 2.00)** | 29.6 (25.9, 33.8) | 1.22 (0.93, 1.62) | 0.80 (0.59, 1.09) |  |
| P-trend | - | **0.005** | - | 0.240 | **-** | **-** |

TyG, triglyceride-glucose index; PP, pulse pressure; HR, hazard ratio; CI, confidence interval; SD, standard deviation.

The **bold font** indicates significance.

**Supplemental Table 11. Associations between triglyceride-glucose index and incident stroke stratified by pulse pressure.**

| **TyG** | **PP < median (51mmHg) (N=3289)** | | **PP ≥ median (51mmHg) (N=3269)** | | **Relative HR with PP group increment** | **P-interaction** |
| --- | --- | --- | --- | --- | --- | --- |
|  | **Incidence rate & 95% CI (Per 1000 person-years)** | **HR (95% CI)** | **Incidence rate & 95% CI (Per 1000 person-years)** | **HR (95% CI)** |  |  |
| Per 1-SD increment | 8.8 (7.8, 10.0) | **1.40 (1.21, 1.62)** | 13.2 (12.0, 14.7) | 1.10 (0.97, 1.25) | **0.86 (0.74, 0.99)** | **0.033** |
| Q1[<8.21] | 4.8 (3.5, 6.5) | Reference | 7.4 (5.6, 9.9) | Reference | Reference | **0.027** |
| Q2[8.21~8.58] | 6.7 (5.1, 8.8) | 1.30 (0.85, 2.00) | 12.8 (10.4, 15.9) | **1.49 (1.03, 2.15)** | 0.98 (0.79, 1.23) |  |
| Q3[8.58~9.02] | 11.0 (8.7, 13.8) | **1.95 (1.29, 2.95)** | 15.5 (12.9, 18.6) | **1.55 (1.07, 2.14)** | 0.85 (0.61, 1.19) |  |
| Q4[>9.02] | 14.7 (11.9, 18.1) | **2.19 (1.40, 3.42)** | 16.3 (13.7, 19.5) | 1.19 (0.80, 1.77) | **0.62 (0.39, 0.99)** |  |
| P-trend | - | **<0.001** | - | 0.694 | **-** | **-** |

TyG, triglyceride-glucose index; PP, pulse pressure; HR, hazard ratio; CI, confidence interval; SD, standard deviation.

The **bold font** indicates significance.

**Supplemental Table 12. Associations between triglyceride-glucose index and incident cardiovascular disease after participants with diabetes mellitus.**

| TyG | **Hazard ratio & 95% confidence interval** | | | | **Relative HR with SBP group increment** | **P-interaction** |
| --- | --- | --- | --- | --- | --- | --- |
|  | **Overall (N=5567)** | **SBP < 120mmHg (N=2161)** | **SBP = 120-129 mmHg (N=1143)** | **SBP ≥ 130mmHg (N=2263)** |  |  |
| **Incident cardiovascular disease** | | | | | | |
| Per 1-SD increment | **1.21 (1.12, 1.31)** | **1.35 (1.17, 1.56)** | **1.34 (1.13, 1.60)** | 1.09 (0.97, 1.22) | **0.88 (0.82, 0.95）** | **0.001** |
| Q1[<8.21] | Reference | Reference | Reference | Reference | Reference | **0.001** |
| Q2[8.21~8.58] | **1.28 (1.08, 1.52)** | 1.32 (0.98, 1.76) | 1.34 (0.92, 1.94) | 1.20 (0.93, 1.55) | 0.95 (0.87, 1.04) |  |
| Q3[8.58~9.02] | **1.49 (1.25, 1.77)** | **1.61 (1.19, 2.19)** | **1.76 (1.21, 2.56)** | 1.27 (0.98, 1.65) | **0.87 (0.76, 0.99)** |  |
| Q4[>9.02] | **1.51 (1.24, 1.84)** | **2.02 (1.43, 2.87)** | **1.72 (1.11, 2.67)** | 1.19 (0.89, 1.59) | **0.75 (0.63, 0.90)** |  |
| P-trend | **<0.001** | **<0.001** | **0.005** | 0.252 | **-** | **-** |

TyG, triglyceride-glucose index; SBP, systolic blood pressure; HR, hazard ratio; CI, confidence interval; SD, standard deviation.

The **bold font** indicates significance.

**Supplemental Figure 1. Study flowchart.**


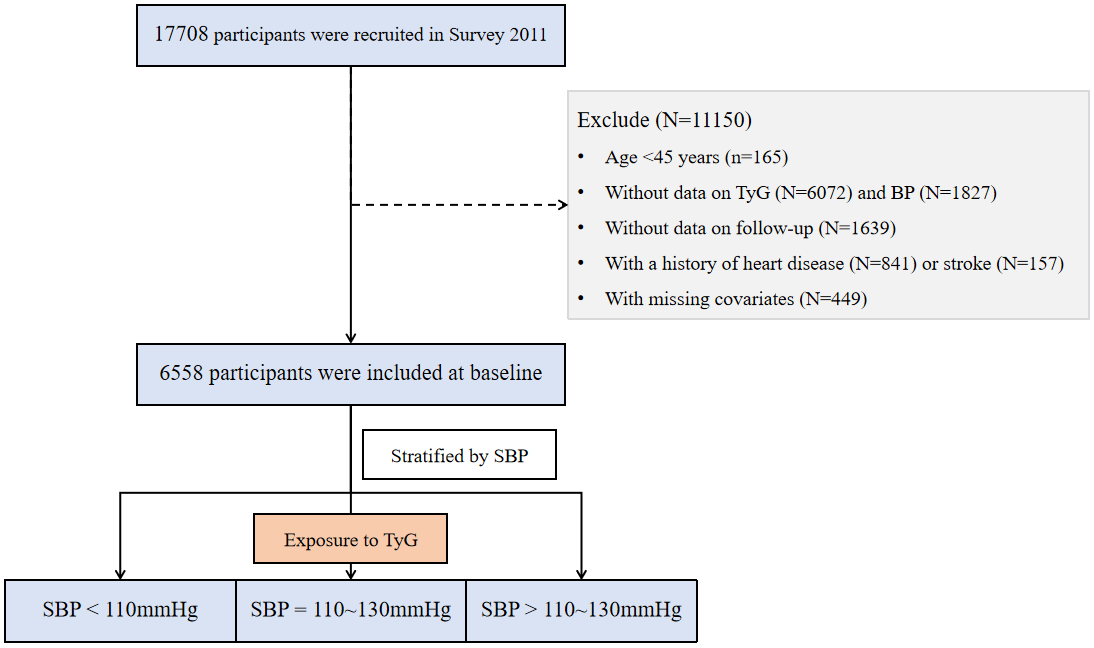


TyG, triglyceride-glucose index; BP, blood pressure; SBP, systolic blood pressure.

**Supplemental Figure 2. Mediated effects of blood pressure on the association between the triglyceride-glucose index with incident heart disease.**


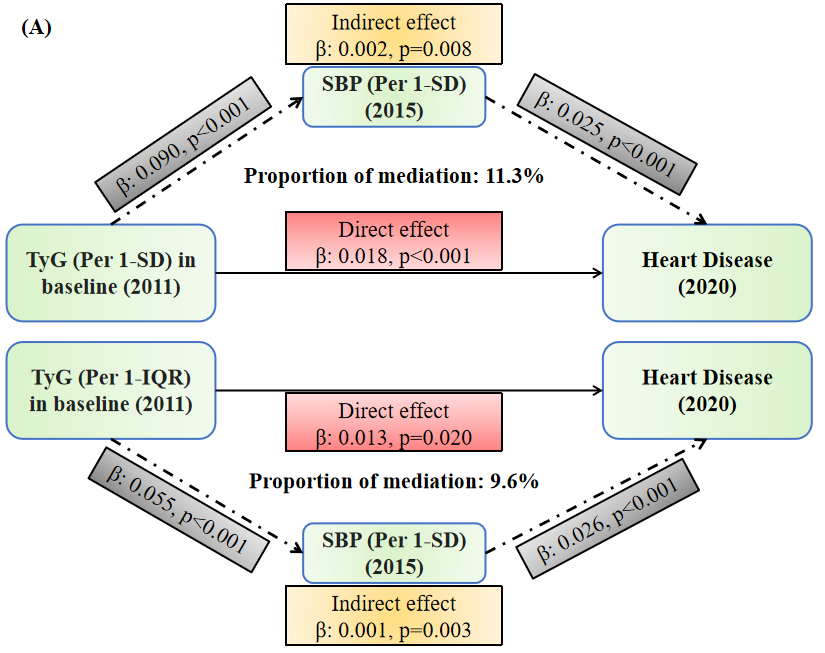


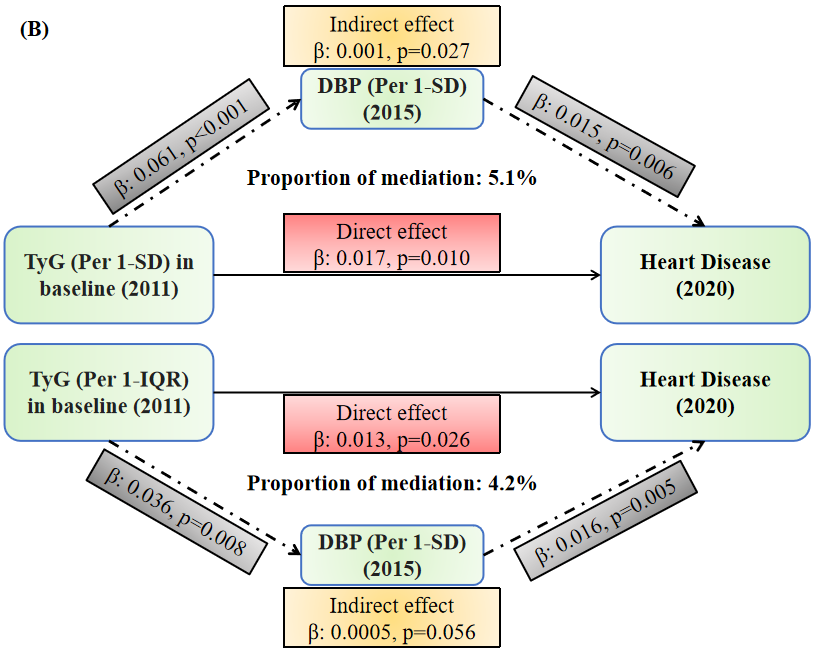


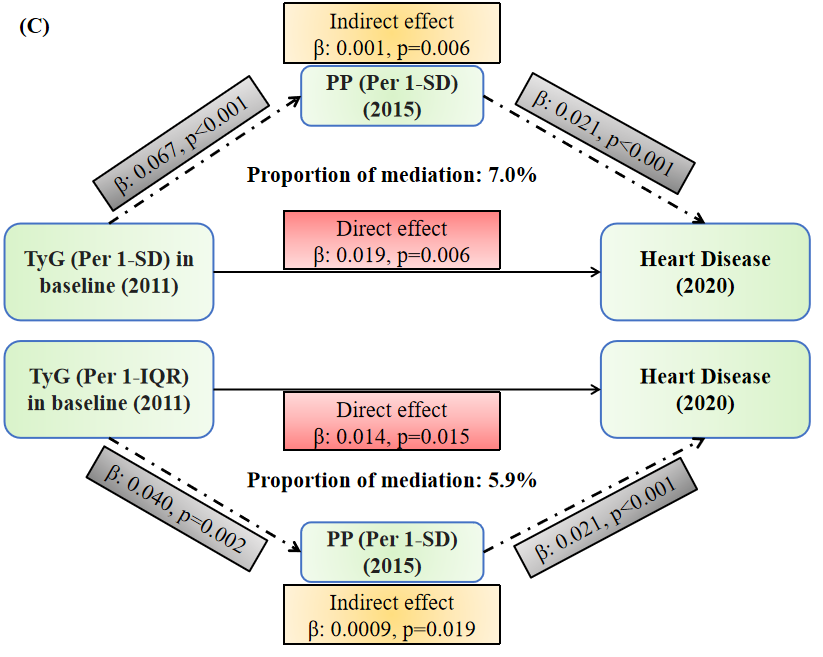


TyG, triglyceride-glucose index; SBP, systolic blood pressure; DBP, diastolic blood pressure; PP, pulse pressure; SD, standard deviation, IQR, interquartile range.

**Supplemental Figure 3. Mediated effects of blood pressure on the association between the triglyceride-glucose index with incident stroke.**


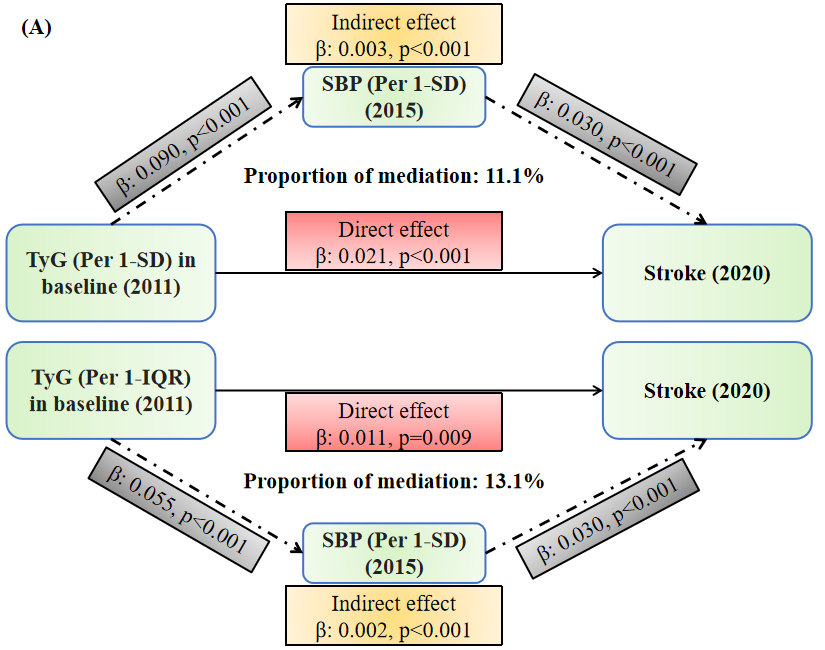


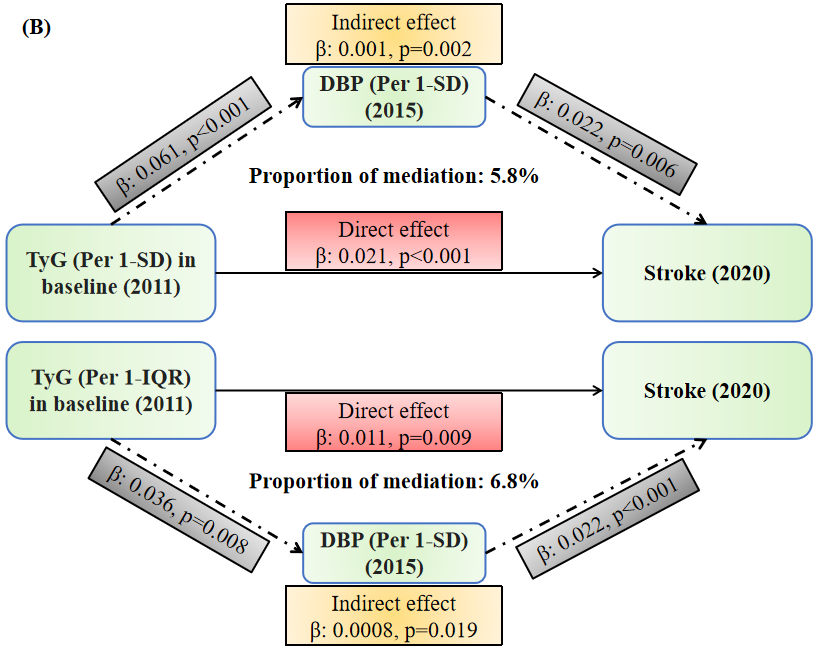


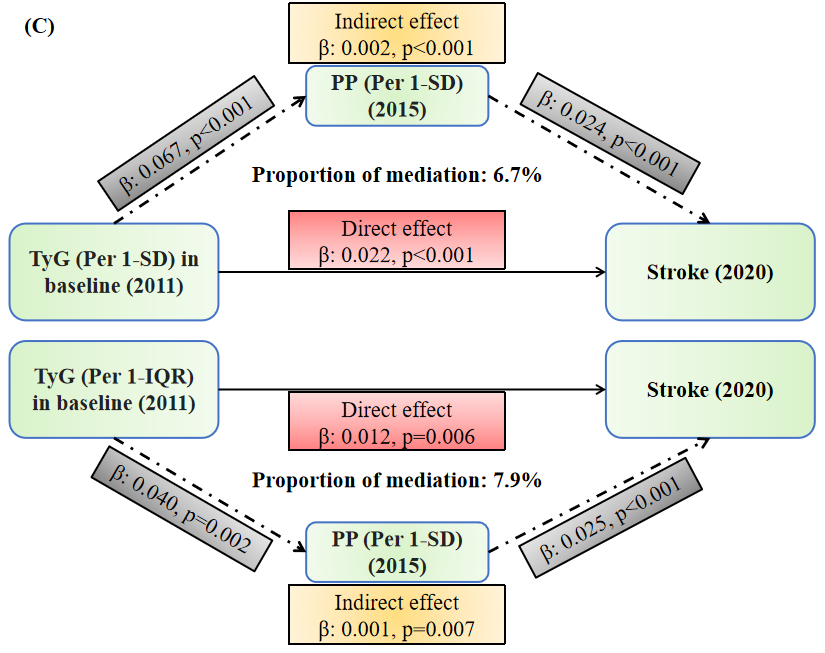


TyG, triglyceride-glucose index; SBP, systolic blood pressure; DBP, diastolic blood pressure; PP, pulse pressure; SD, standard deviation, IQR, interquartile range.

**Supplemental Figure 4. Associations between triglyceride-glucose index and risk probability of incident heart disease (A) and stroke (B) stratified by systolic blood pressure.**


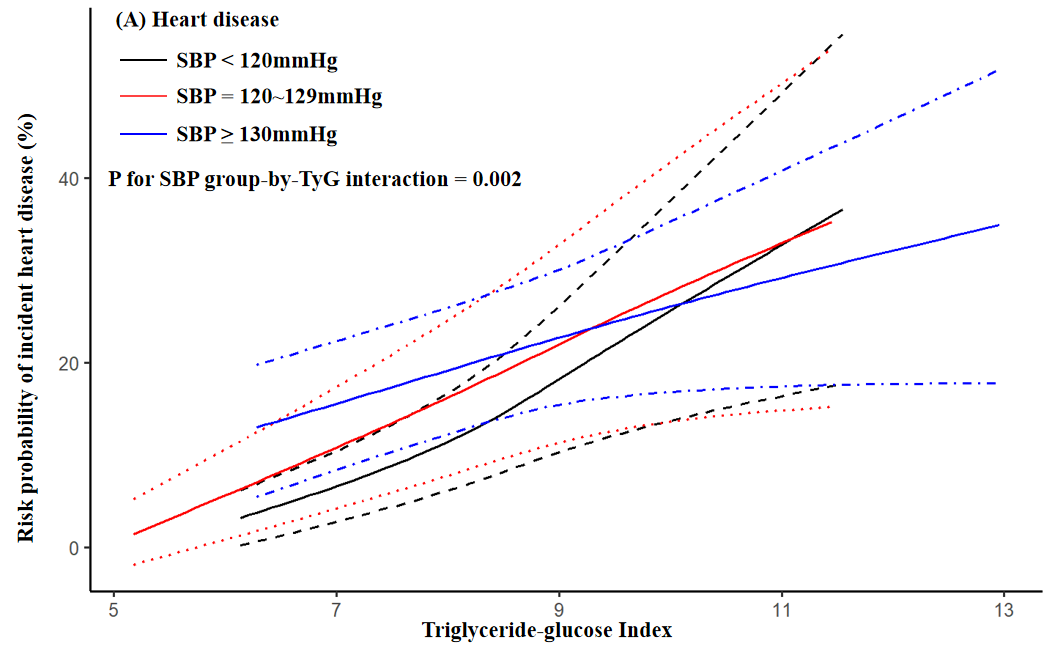

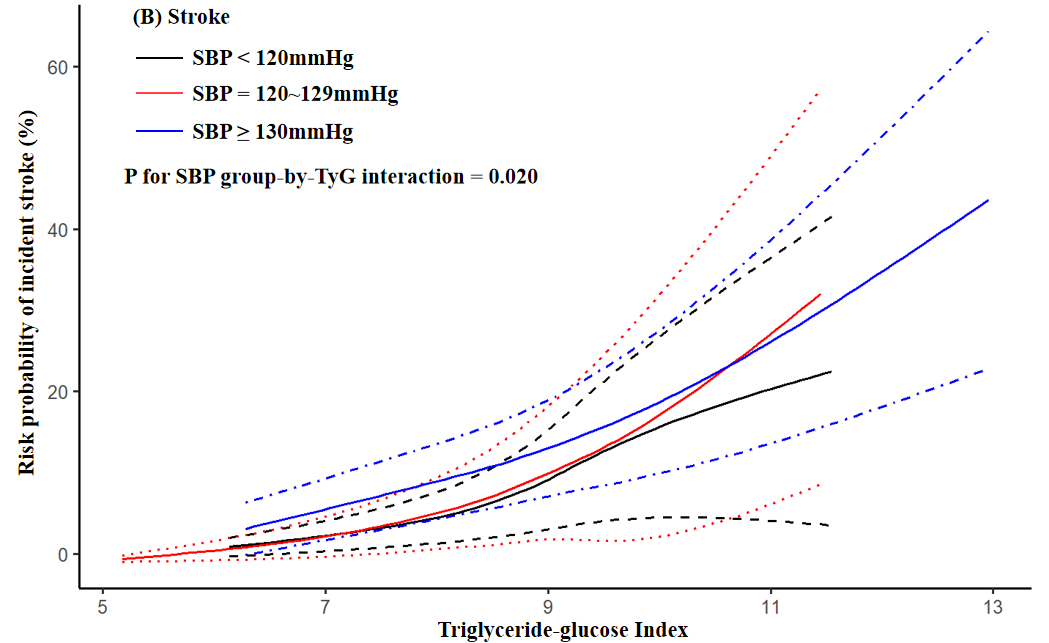


SBP, systolic blood pressure; TyG, triglyceride-glucose index.

**Supplemental Figure 5. Restricted cubic spline analysis of triglyceride-glucose index with incident heart disease stratified by systolic blood pressure.**


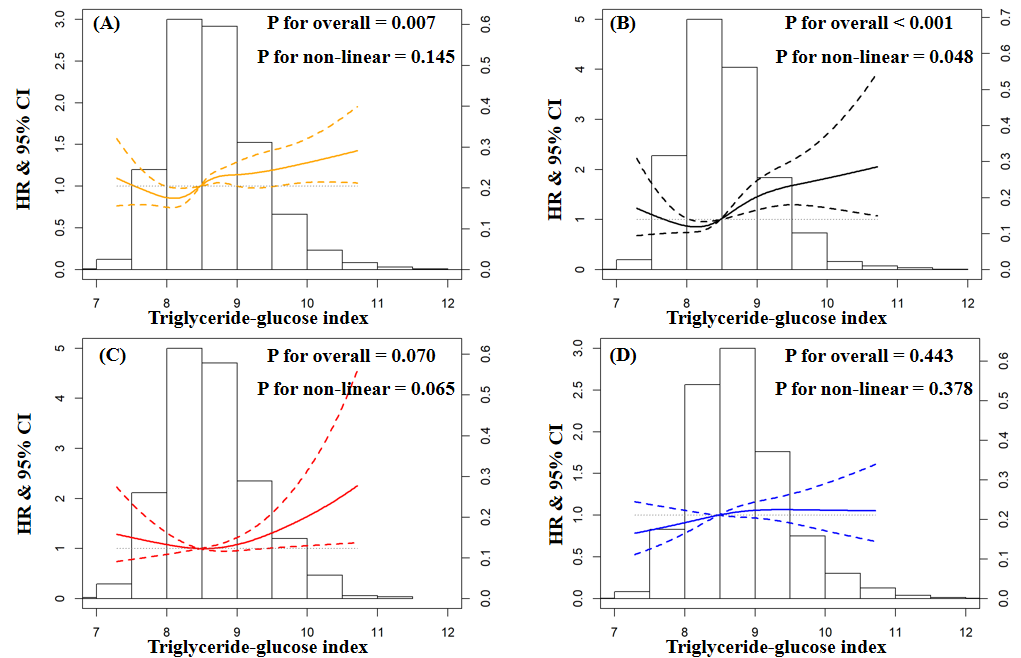


Panel A: Non-linear relationship between TyG and incident heart disease among overall participants.

Panel B: Non-linear relationship between TyG and incident heart disease among participants with SBP < 120mmHg.

Panel C: Non-linear relationship between TyG and incident heart disease among participants with SBP between 120 and 129mmHg.

Panel D: Non-linear relationship between TyG and incident heart disease among participants with SBP ≥ 130mmHg.

HR hazard ratio; CI, confidence interval.

**Supplemental Figure 6. Restricted cubic spline analysis of triglyceride-glucose index with incident stroke stratified by systolic blood pressure.**


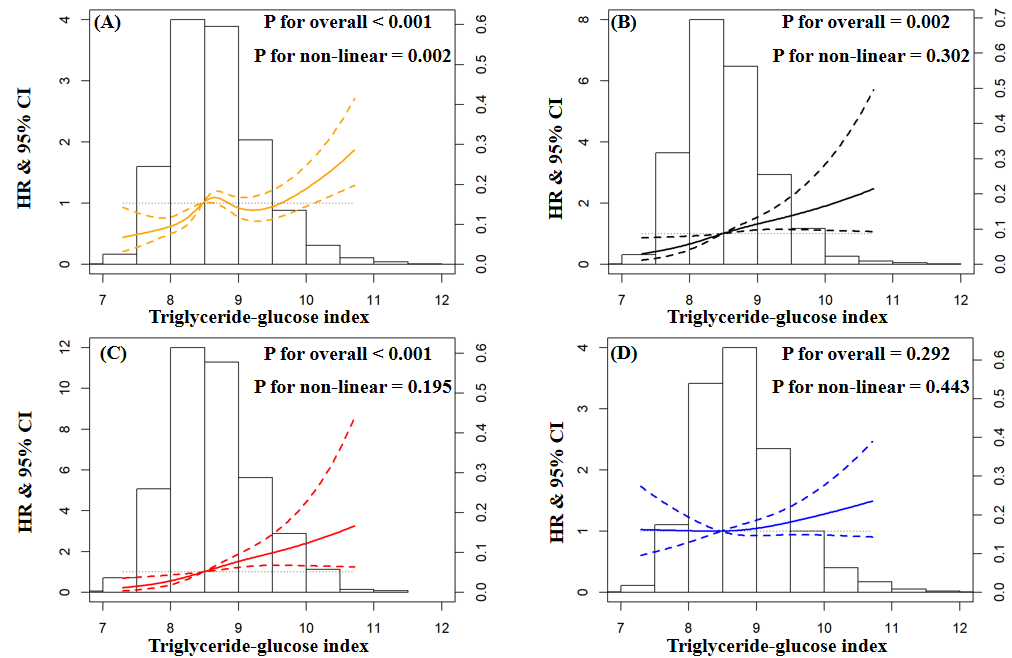


Panel A: Non-linear relationship between TyG and incident stroke among overall participants.

Panel B: Non-linear relationship between TyG and incident stroke among participants with SBP < 120mmHg.

Panel C: Non-linear relationship between TyG and incident stroke among participants with SBP between 120 and 129mmHg.

Panel D: Non-linear relationship between TyG and incident stroke among participants with SBP ≥ 130mmHg.

HR hazard ratio; CI, confidence interval.

**Supplemental Figure 7. Relationships between triglyceride-glucose index and risk probability of incident cardiovascular disease (A), heart disease (B), and stroke (C) stratified by diastolic blood pressure.**


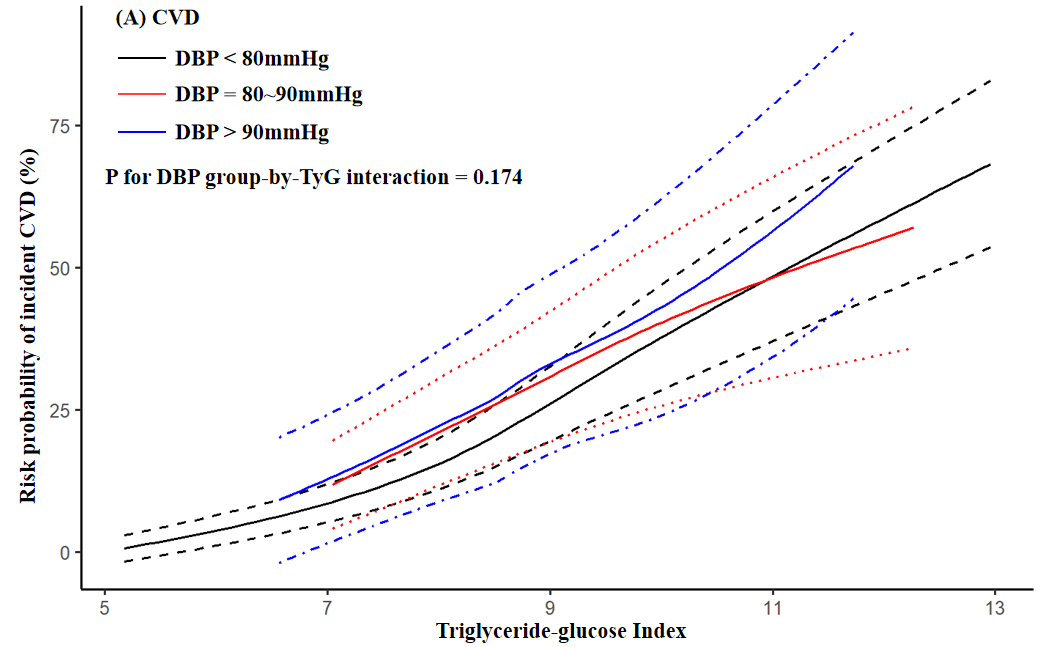

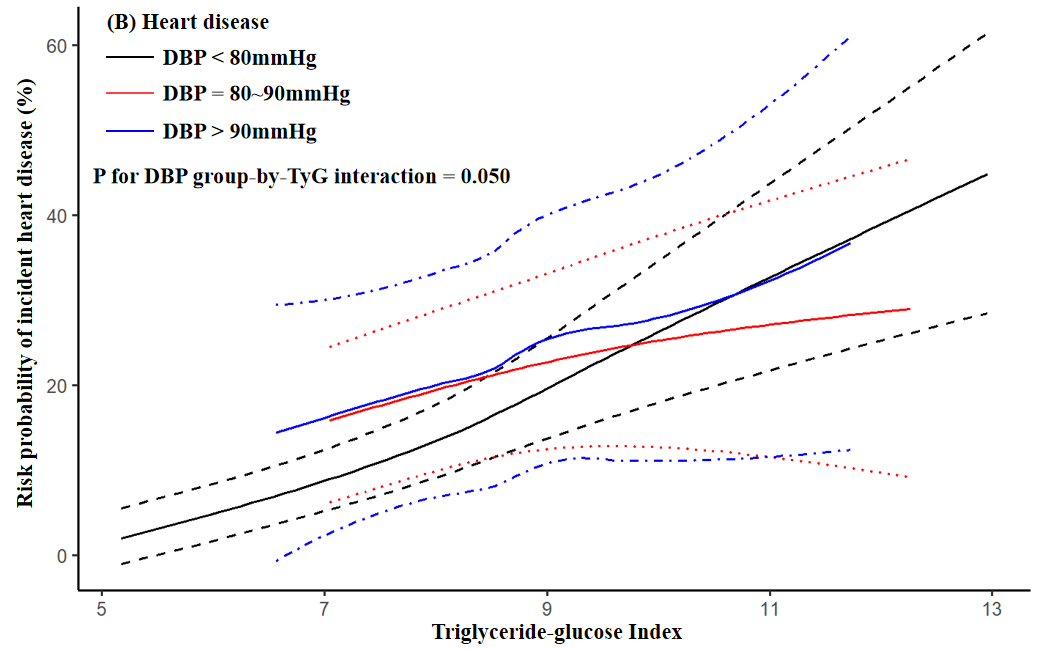

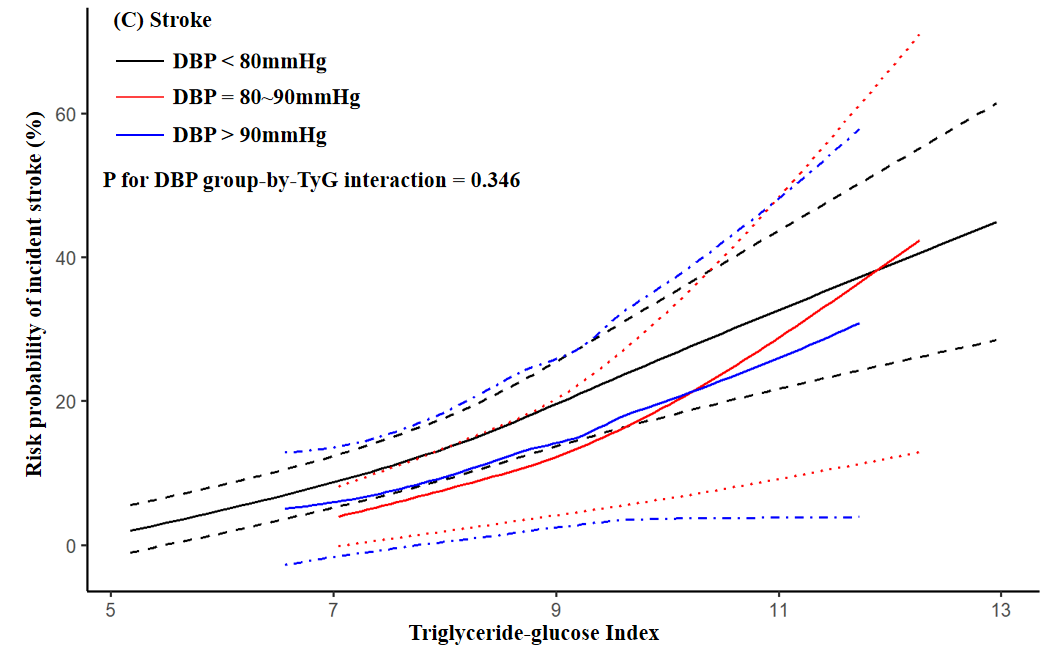


CVD, cardiovascular; DBP, diastolic blood pressure; TyG, triglyceride-glucose index.

**Supplemental Figure 8. Restricted cubic spline analysis of triglyceride-glucose index with incident cardiovascular disease stratified by diastolic blood pressure.**


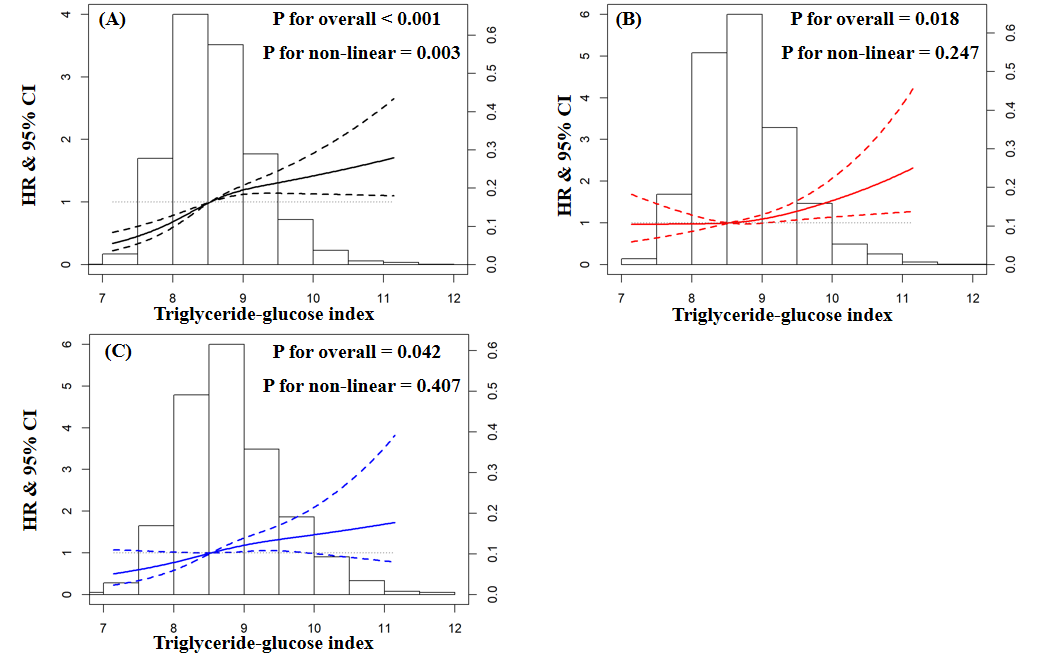


Panel A: Non-linear relationship between TyG and incident CVD among participants with DBP < 80mmHg.

Panel B: Non-linear relationship between TyG and incident CVD among participants with DBP between 80 and 90mmHg.

Panel C: Non-linear relationship between TyG and incident CVD among participants with DBP > 90mmHg.

HR hazard ratio; CI, confidence interval.

**Supplemental Figure 9. Restricted cubic spline analysis of triglyceride-glucose index with incident heart disease stratified by diastolic blood pressure.**


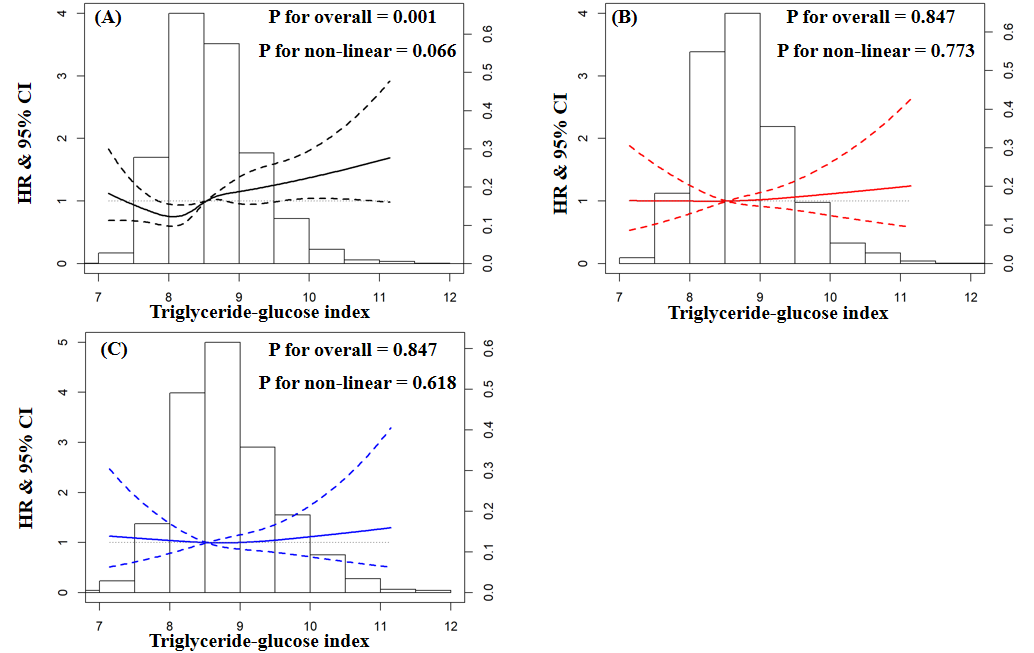


Panel A: Non-linear relationship between TyG and incident heart disease among participants with DBP < 80mmHg.

Panel B: Non-linear relationship between TyG and incident heart disease among participants with DBP between 80 and 90mmHg.

Panel C: Non-linear relationship between TyG and incident heart disease among participants with DBP > 90mmHg.

HR hazard ratio; CI, confidence interval.

**Supplemental Figure 10. Restricted cubic spline analysis of triglyceride-glucose index with incident stroke stratified by diastolic blood pressure.**


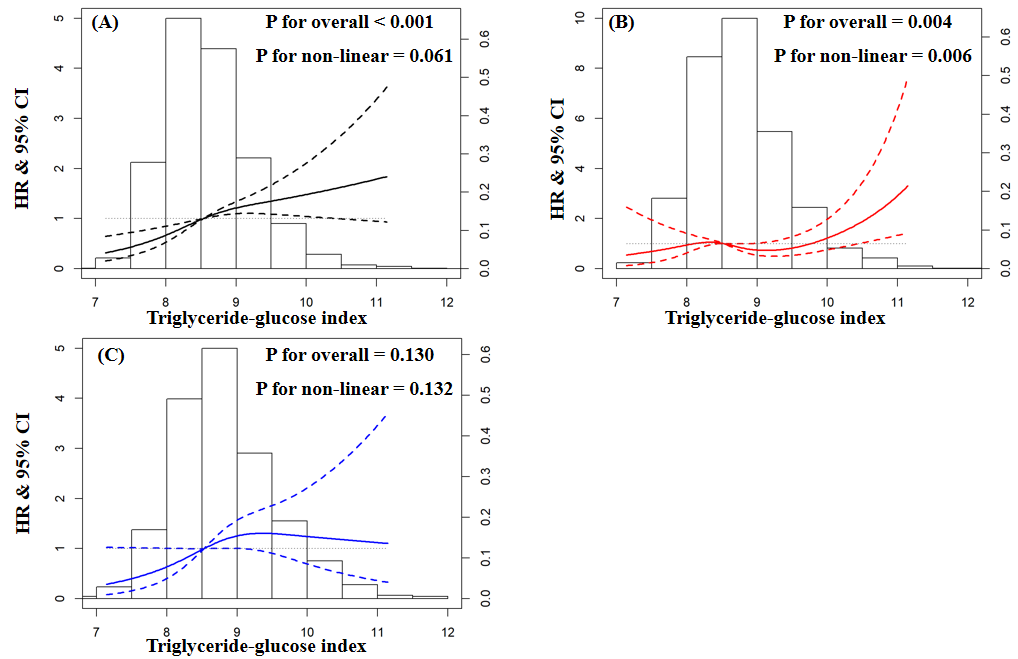


Panel A: Non-linear relationship between TyG and incident stroke among participants with DBP < 80mmHg.

Panel B: Non-linear relationship between TyG and incident stroke among participants with DBP between 80 and 90mmHg.

Panel C: Non-linear relationship between TyG and incident stroke among participants with DBP > 90mmHg.

HR hazard ratio; CI, confidence interval.

**Supplemental Figure 11. Associations between triglyceride-glucose index and risk probability of incident cardiovascular disease (A), heart disease (B), and stroke (C) stratified by pulse pressure.**


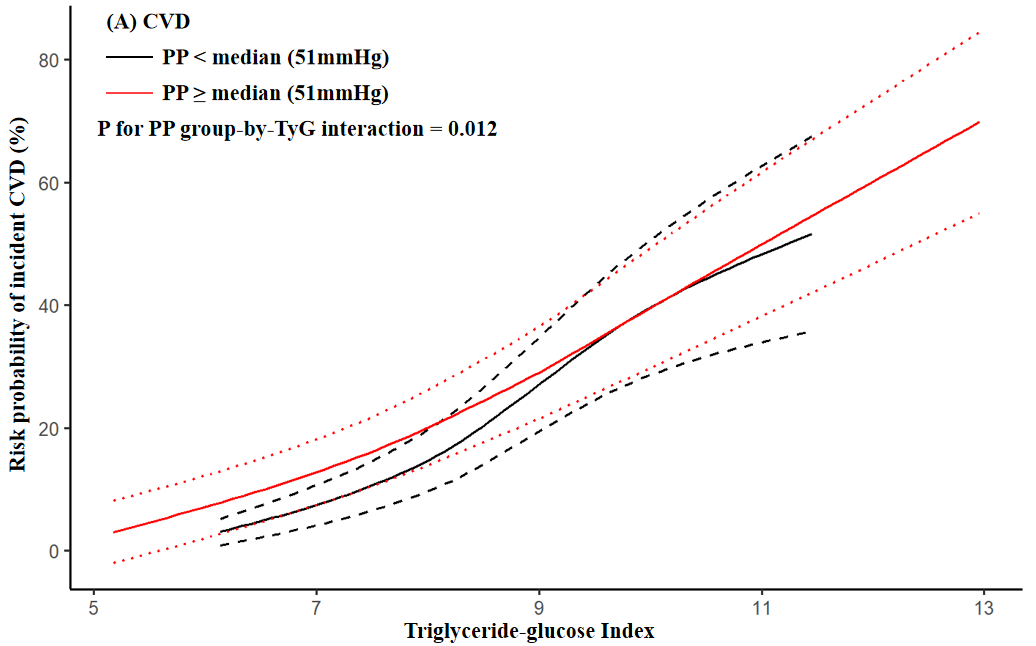

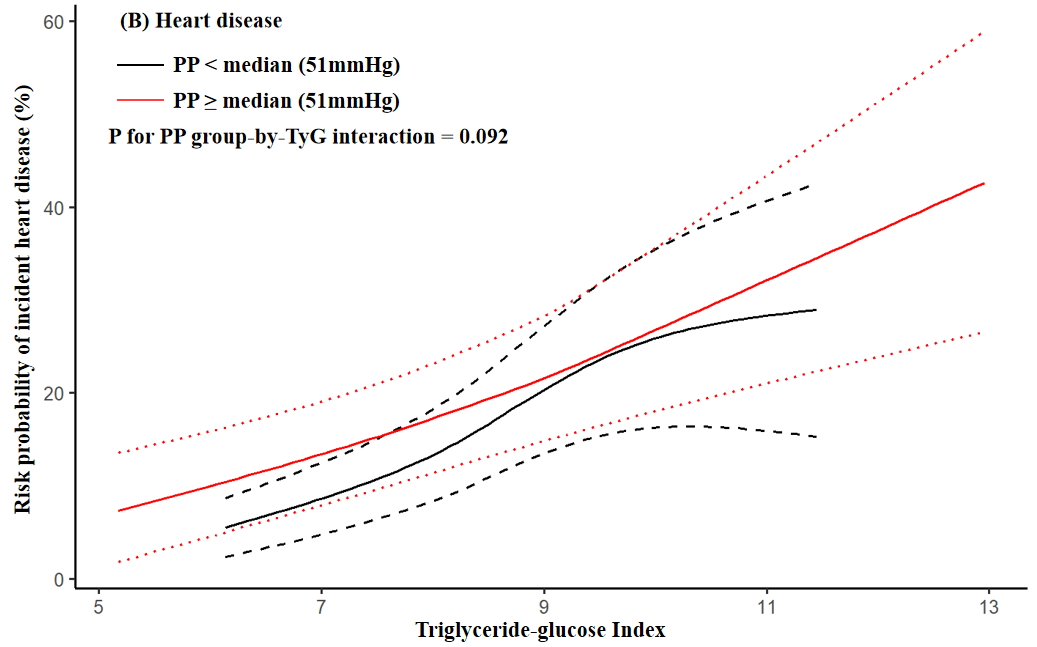

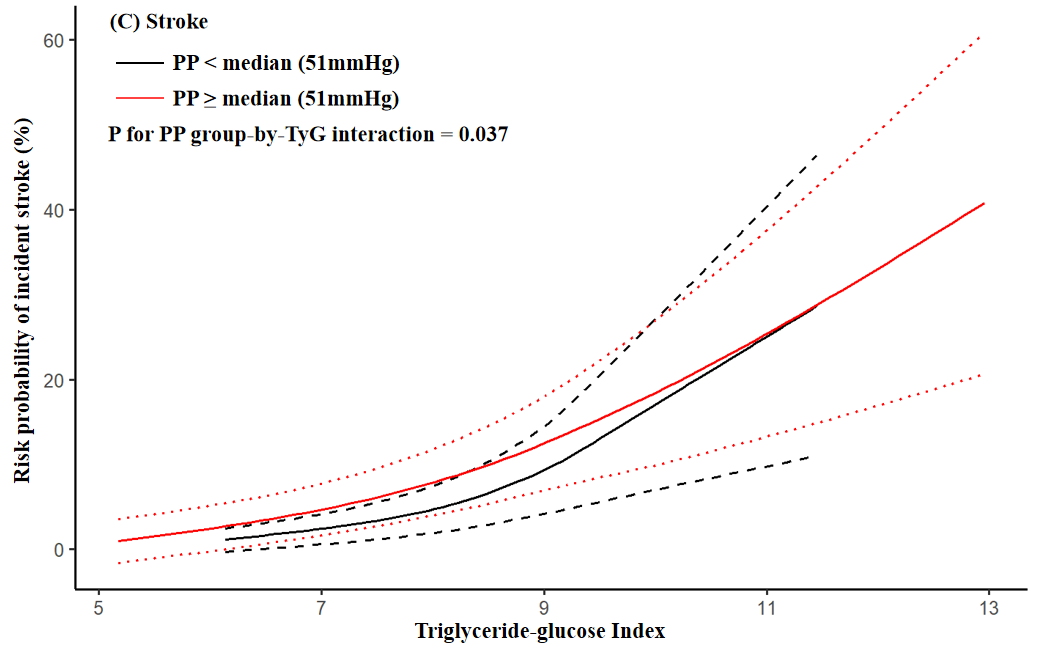


CVD, cardiovascular; PP, pulse pressure; TyG, triglyceride-glucose index.

**Supplemental Figure 12. Restricted cubic spline analysis of triglyceride-glucose index with incident cardiovascular disease stratified by pulse pressure.**


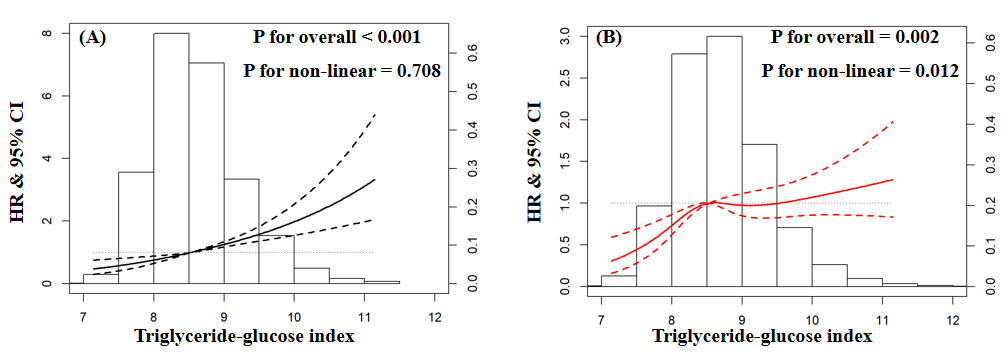


Panel A: Non-linear relationship between TyG and incident CVD among participants with PP < median (51mmHg).

Panel B: Non-linear relationship between TyG and incident CVD among participants with PP ≥ median (51mmHg).

HR hazard ratio; CI, confidence interval.

**Supplemental Figure 13. Restricted cubic spline analysis of triglyceride-glucose index with incident heart disease stratified by pulse pressure.**


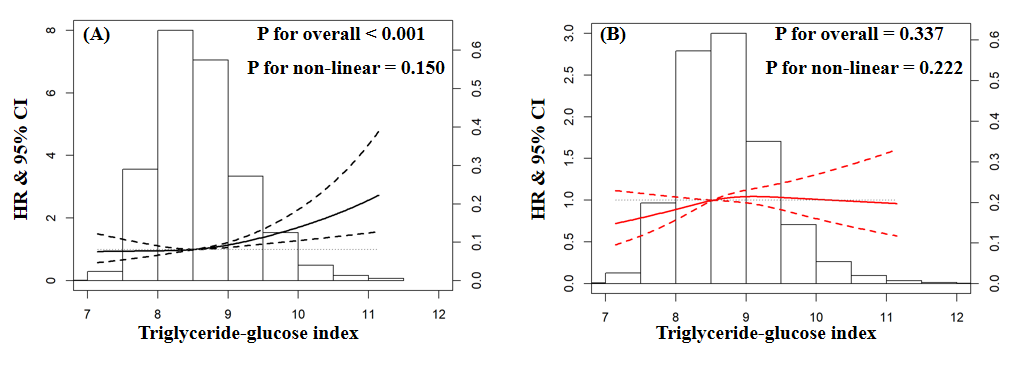


Panel A: Non-linear relationship between TyG and incident heart disease among participants with PP < median (51mmHg).

Panel B: Non-linear relationship between TyG and incident heart disease among participants with PP ≥ median (51mmHg).

HR hazard ratio; CI, confidence interval.

**Supplemental Figure 14. Restricted cubic spline analysis of triglyceride-glucose index with incident stroke stratified by pulse pressure.**


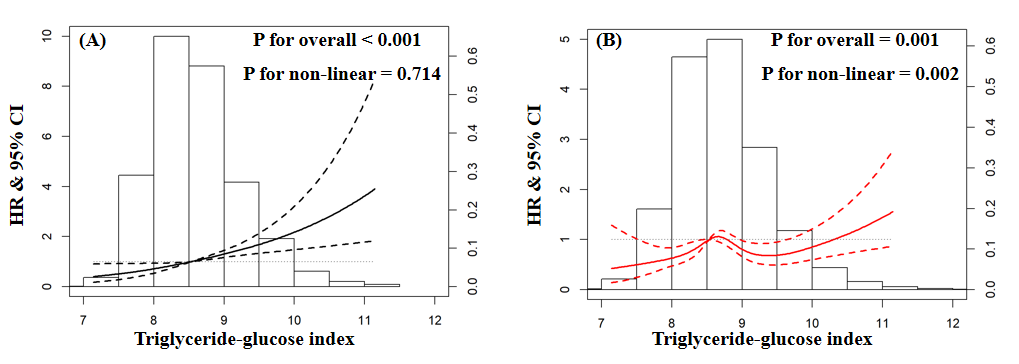


Panel A: Non-linear relationship between TyG and incident stroke among participants with PP < median (51mmHg).

Panel B: Non-linear relationship between TyG and incident stroke among participants with PP ≥ median (51mmHg).

HR hazard ratio; CI, confidence interval.
